# Mixing chromatin fibers with different nucleosome repeat lengths changes dynamics of chromatin phase separation

**DOI:** 10.1016/j.jbc.2026.113386

**Published:** 2026-08-11

**Authors:** Jeremy Ernst, Beatriz Orozco Monroy, John van Noort

**Affiliations:** Biological and Soft Matter Physics, Huygens-Kamerlingh Onnes Laboratory, Leiden University, Leiden, Netherlands

**Keywords:** chromatin, chromatin structure, chromatin regulation, fluorescence, phase-separation, nucleosome, nucleosome repeat length

## Abstract

The eukaryotic genome is organized into chromatin at multiple lengths and timescales. Liquid-liquid phase separation has recently emerged as a mechanism for the dynamic compartmentalization of chromatin. However, it remains unclear how cells can locally alter phase separation behavior to condense, decondense, and segregate specific regions of their genome. Selective interactions between chromatin fibers with different nucleosome repeat lengths (NRLs), as well as their incorporation into existing condensates composed of different NRL chromatin fibers, may provide a pathway for such processes. Using fluorescence microscopy, we investigated how these mechanisms influence the formation, coalescence, and maturation of chromatin condensates. Our results show distinct NRL-dependent mixing behaviors of chromatin before and after condensate formation. 167 and 197 NRL fibers, known to fold into compact fibers by strong nucleosome stacking interactions, formed amorphous condensates. In contrast, 172 and 202 NRL fibers, which only allow for weak stacking, formed spherical condensates. When NRLs were mixed, amorphous condensates exhibited localized clustering of identical NRLs. In spherical condensates, however, both NRLs were homogeneously distributed, with a varying NRL ratio per condensate. In addition, incorporation of 167 NRLs into preexisting 172 NRL condensates resulted in a multiphase structure where 167 NRL fibers formed an outer layer. These findings present an intrinsic link between DNA sequence, nucleosome positioning, local chromatin configuration and multiscale phase separation behavior. More broadly, they contribute to a deeper understanding of the dynamic methods of genome organization employed by eukaryotic organisms.

The eukaryotic genome is compacted and organized at multiple scales. At the smallest scale, 147 bps of DNA wrap around an octamer core of two copies of the four histone proteins H2A, H2B, H3, and H4 to form a nucleosome (1, 2). These DNA–histone interactions are mediated by positively charged arginine and lysine residues of the histones and the negatively charged phosphate backbone of DNA (3, 4). Nucleosomes are spaced by linker DNA, of which the length can vary between chromatin loci, tissues, and organisms (5, 6, 7). At the next level of compaction, nucleosomes interact through stacking interactions between the H4 N-terminal tail and the negative charges in the H2A/H2B acidic patch of neighboring nucleosomes (1, 8, 9, 10, 11). These interactions result in a higher-order chromatin structure referred to as the chromatin fiber (12, 13). The structure of the chromatin fiber can be influenced by posttranslational modifications (14), the presence of linker histones (15), or other chromatin-associated proteins (16, 17).

At the larger scale, chromatin is compartmentalized into topologically associating domains (TADs) (18, 19, 20). TADs vary in local compaction, with distinct transcriptionally active euchromatin regions of low density and loose packaging of nucleosomes, as well as high nucleosome density, transcriptionally silent heterochromatin regions (21). Chromosomes are made up of multiple TADs, and are spatially constrained in their own chromosome territories (22, 23). It has become clear that this organization is not merely hierarchical: changes in local nucleosome conformation can affect chromatin fiber structure (24), while chromatin fiber structure influences the accessibility of nucleosomal DNA to DNA-binding proteins such as transcription factors (25, 26).

The understanding of the chromatin fiber’s structure has changed in recent years. Initial studies suggested a compact “30 nm fiber” with nucleosomes stacked in a superhelical topology (13, 27, 28). Recent studies have, however, questioned the *in vivo* relevance of this model (29, 30, 31, 32, 33). There are instead strong indications for a looser ’beads on a string’ configuration that coalesces into larger supramolecular condensates (34, 35). Regardless, chromatin fiber organization is a highly dynamic (36, 37), yet regulated process (38).

The proposed mechanism for the formation of chromatin condensates is liquid-liquid phase separation (LLPS). In the LLPS of biomolecules, weak multivalent interactions between molecules allow for the formation of highly concentrated compartments (39, 40). Chromatin has long been known to aggregate at sufficiently strong ionic conditions (35, 41, 42), but a full description of its phase-separation behavior has not yet emerged. In one of the seminal works on chromatin phase separation, Gibson *et al.* (43) demonstrated that inter-nucleosome histone-tail stacking interactions mediate LLPS of chromatin. In many *in vitro* studies such as this one, the 147 bps Widom 601 sequence is used for its strong nucleosome positioning affinity (44), allowing for perfect control over inter-nucleosome spacing (45, 46). This spacing, in turn, has been shown to influence gene activity (47), as well as being a determining factor in chromatin LLPS (43).

Oswald ripening allows condensate growth through absorption of material from the surrounding solution, increasing the solubility of fibers within the aggregate relative to the bulk (48, 49). In addition, LLPS aggregates grow through coalescence, where two condensates merge to form a larger condensate, resulting in an energetically favorable reduction of surface tension. Studying the merging and ripening behavior of different chromatin condensates can therefore provide insight into the state of phase-separated chromatin, for which both theoretical frameworks and simulation-based models have now also been established (50, 51, 52, 53). Previous research on liquid-like (54, 55) and solid-like (56) behavior of chromatin condensates revealed an influence of crowding agents such as bovine serum albumin (BSA) and acetate anions on condensate formation.

Though LLPS of chromatin is now well-established, both *in vitro* and *in vivo*, it remains an open question how eukaryotic cells regulate the formation of condensates for particular loci. One mechanism could be to arrange nucleosome spacing by remodelers to alter the nucleosome repeat length (NRL) for specific loci, thereby changing the susceptibility to LLPS. Here, we investigated how chromatin fibers of different NRLs are incorporated into existing condensates. We compared results for NRLs that were mixed before and after phase separation was induced. These results provide insight into the effects of heterogeneous NRLs precondensate and postcondensate formation, which may be important for *in vivo* chromatin organization and its role in transcription regulation.

## Results

**Figure 1:**
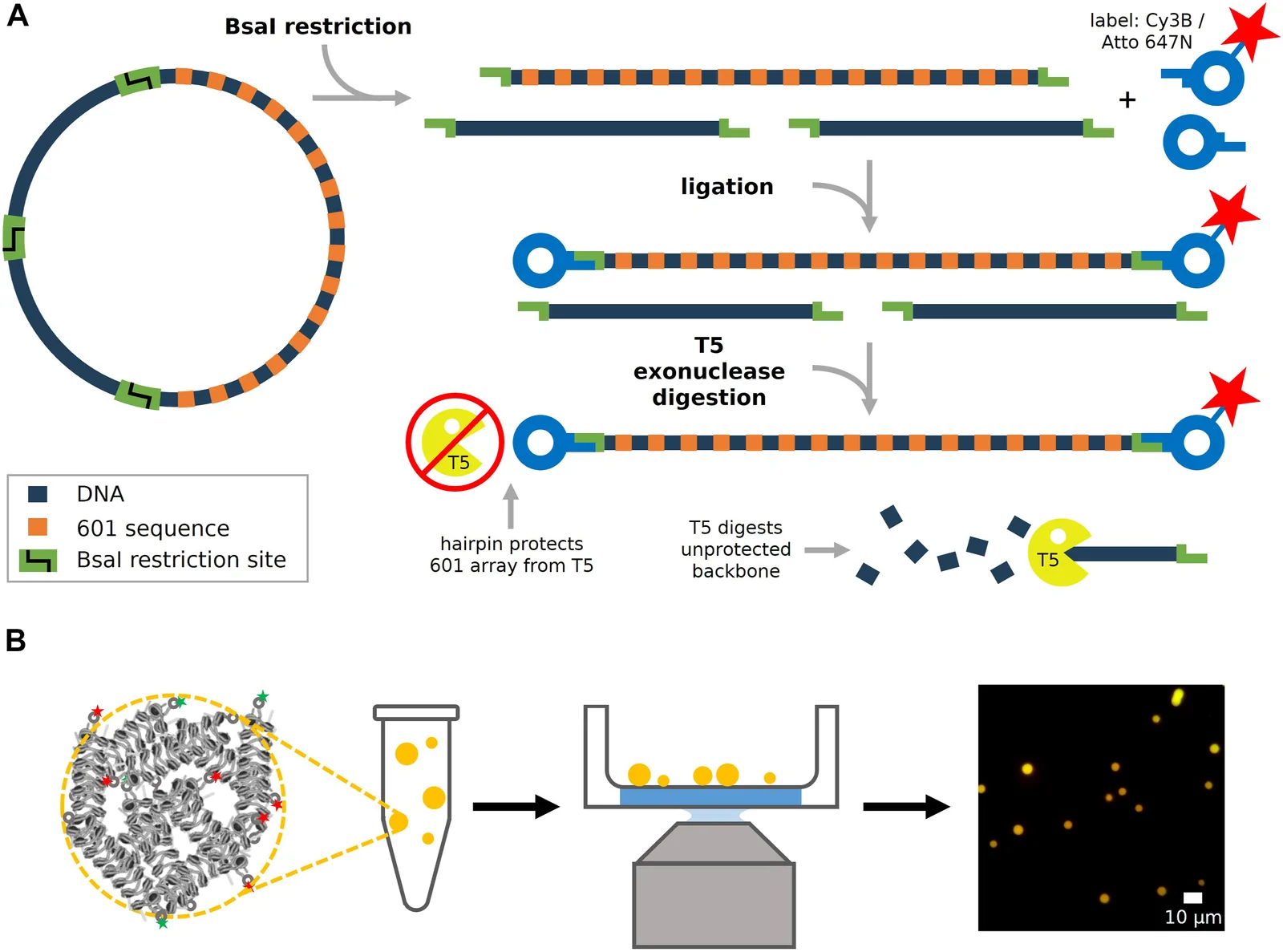
**DNA substrates for chromatin phase separation studies were created using a novel approach combining Golden Gate assembly, DNA hairpins, and exonuclease treatment.***A*, plasmids containing arrays of Widom 601 sequences (*orange*) were digested at three unique BsaI restriction sites (*green*). DNA hairpins (*blue*) with optional fluorophore labels (*red*), complementary to BsaI restriction sites, were ligated to the 601 arrays, which protected them from the T5 exonuclease. *B*, Cy3B and Atto 647N-labeled 601 arrays were reconstituted into chromatin fibers. Varying stoichiometries of labeled and nonlabeled fibers with different NRLs were left to sediment to the bottom of the multiwell imaging plate and subsequently imaged using multicolor fluorescence microscopy. Schematic of chromatin fiber adapted from (36). NRL, nucleosome repeat length.

One of the driving factors in chromatin phase separation is a high nucleosome concentration (43). We therefore developed a method, making use of Golden Gate assembly, for producing large amounts of DNA containing various NRLs without requiring gel purification (Fig. 1*A*). To do so, three recognition sites for the restriction enzyme BsaI were introduced in a plasmid containing the desired number of repeats of the Widom 601 nucleosome positioning sequence (44). One was in the backbone, and one was on each end of the substrate. DNA hairpins, targeting the ends of the array of 601 nucleosome positioning elements, were included in the Golden Gate assembly reaction. The hairpins optionally contained an attached Cy3B or Atto 647N fluorescent label. The hairpins protect the substrate from T5 exonuclease, which was added after the Golden Gate assembly to degrade the plasmid backbone and excess hairpins, leaving only the desired substrate (Fig. S1*A*). Finally, these substrates were reconstituted into chromatin through salt gradient dialysis with recombinant human histone octamers (Fig. S1*B*).

Phase separation was induced by the addition of a phase separation buffer (final salt concentration 100 mM NaCl, 10 mM MgCl_2_, unless otherwise specified). After a 72 h incubation, the condensates were transferred to a glass-bottomed multiwell plate. Sufficiently large condensates (>1 μm) sedimented and stuck to the surface. These were visualized after 3 h with multicolor fluorescence microscopy (Fig. 1*B*).

### Linker length influences chromatin phase separation behavior

NRL is known to play an important role in chromatin phase separation (43, 54). We therefore investigated the effects of the NRL-dependent phase-separation behavior. NRL refers to the sum of the length (in base pairs) of the Widom 601 nucleosome positioning sequence and the length of the linker DNA that connects it to the next 601 sequence. Each DNA substrate contained 16 repeats of its NRL. We chose 167 and 197 bp NRL chromatin fibers, using 147 bp of the Widom 601 with 20 and 50 bps of linker DNA. These linker lengths have been shown to fold into dense chromatin fibers, driven by stacking interactions between nearest neighbor (197 NRL, a “1-start” topology) (57) and next-nearest neighbor (167 NRL, a “2-start” topology) (36) nucleosomes. In addition, we measured 172 and 202 NRL chromatin fibers, in which energetically unfavorable conformations of the linker DNA inhibit stacking interactions within the fiber (58). The absence of nucleosome stacking in cis, allows for stacking interactions with nucleosomes in other chromatin fibers. These trans-stacking interactions may then mediate the phase separation of the chromatin.

**Figure 2:**
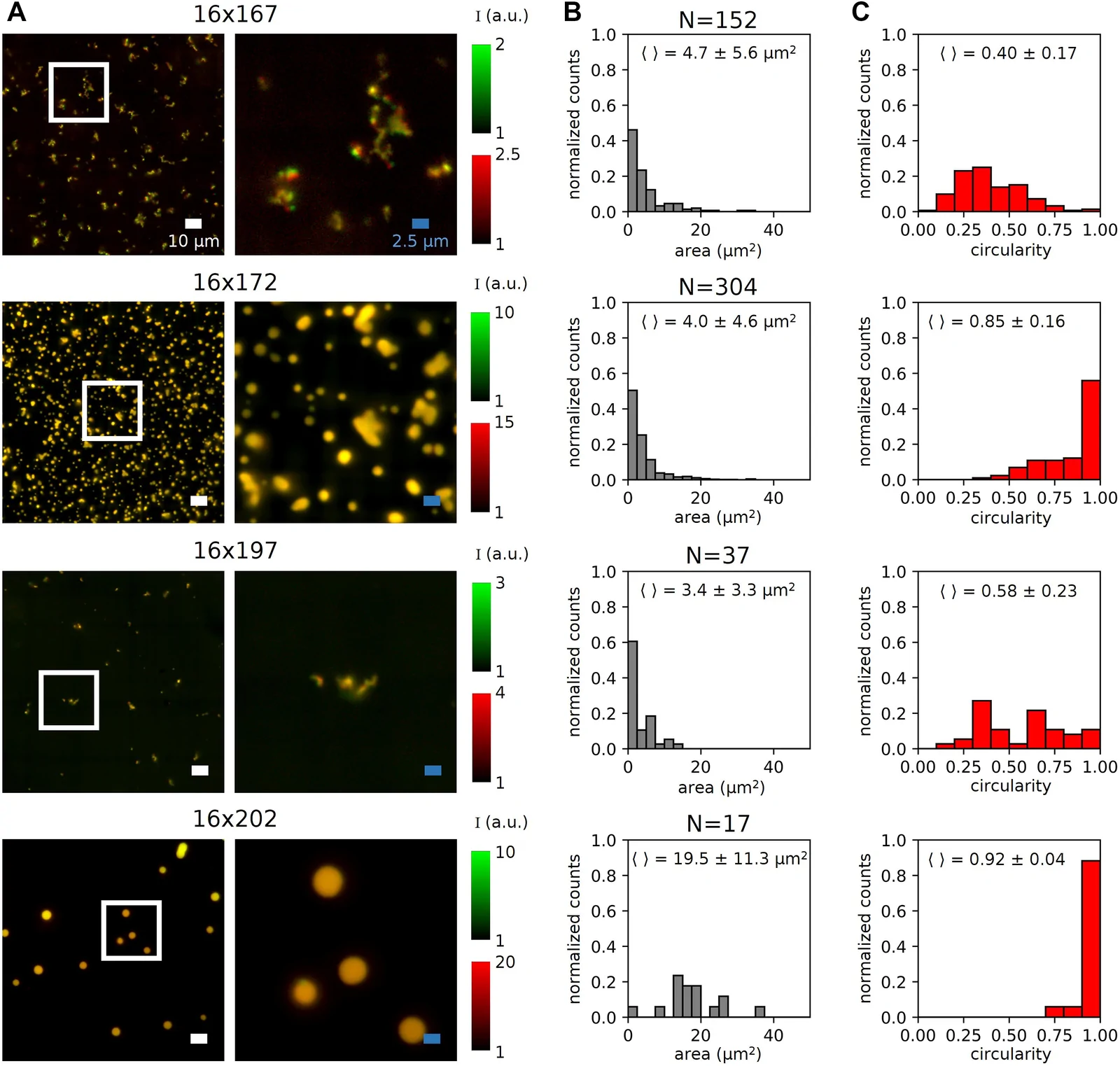
**Chromatin fibers that favor internal stacking form amorphous condensates, while fibers that disfavor it form spherical condensates.***A*, aggregation and phase-separation behavior of chromatin fibers with varying nucleosome repeat lengths (NRL). The *left column* shows the full field of view, the *right column* shows 4× magnification of the insert. The Cy3B-labeled fibers (*green*) and Atto 647N-labeled fibers (*red*) colocalize. *B*, the relative size of condensates. 202 NRL condensates are considerably larger but fewer in number. N is the number of analyzed condensates. *C*, the circularity of condensates distinguishes between amorphous (*asymmetrical*) aggregates (167, 197 NRL) and spherical (*circular*) condensates (172, 202 NRL). Averages ⟨⟩ are shown as the mean ± SD. The scale bars represent 10 μm (*white*) and 2.5 μm (*blue*).

Figure 2*A* shows chromatin fiber aggregation in high mono- and divalent salt concentrations, known to facilitate nucleosome stacking interactions (59, 60, 61, 62). Phase separation behavior depends on both chromatin and salt concentration (Fig. S4). To verify that there was sufficient time for mixing the fibers, we first combined two fractions of chromatin fibers of the same NRL, with 5% of the molecules labeled with Cy3B and 5% with Atto 647N. Since both colored fibers are identical, except for the fluorophore, we expect perfect colocalization of both channels. Indeed, we did not observe variations in the composition of the condensate. We did, however, observe a distinct difference in the morphology of the condensates depending on NRL. The 172 and 202 NRL chromatin formed spherical condensates, indicating liquid-like properties at formation (Fig. 2*A*, second and fourth row). The 202 NRL condensates were larger and less abundant than the 172 NRL condensates. The 167 and 197 NRL chromatin instead formed asymmetrical aggregates (Fig. 2*A*, first and third row), with the 197 aggregates being smaller but similarly irregular, pointing to a solid-like assembly. Note that small shifts in colocalization of the 167 NRL aggregates are most likely due to the movement of the aggregate between the acquisition of the Cy3B and Atto 647N images, suggesting that these are not tightly bound to the surface, presumably due to their irregular shape. The spherical 172 and 202 NRL condensates did not exhibit this behavior.

As a quantification of the size and morphology of the condensates, the distributions of condensate surface area (Fig. 2*B*) and circularity (Fig. 2*C*) are shown. These confirm the difference in aggregation behavior, with an expected circularity of one for the spherical condensates and a smaller circularity for the amorphous condensates. In addition, fluorescence recovery after photobleaching (FRAP) measurements were performed to quantify fiber mobility inside these condensates just after phase separation (Fig. S5) and after 72 h (Fig. S6). In both cases, no significant recovery was observed for any of the measured NRLs. This suggests that condensates that were initially in a liquid-like state had transitioned to a solid-like state by the time they were imaged.

### Mixing NRLs leads to heterogeneous condensates

**Figure 3:**
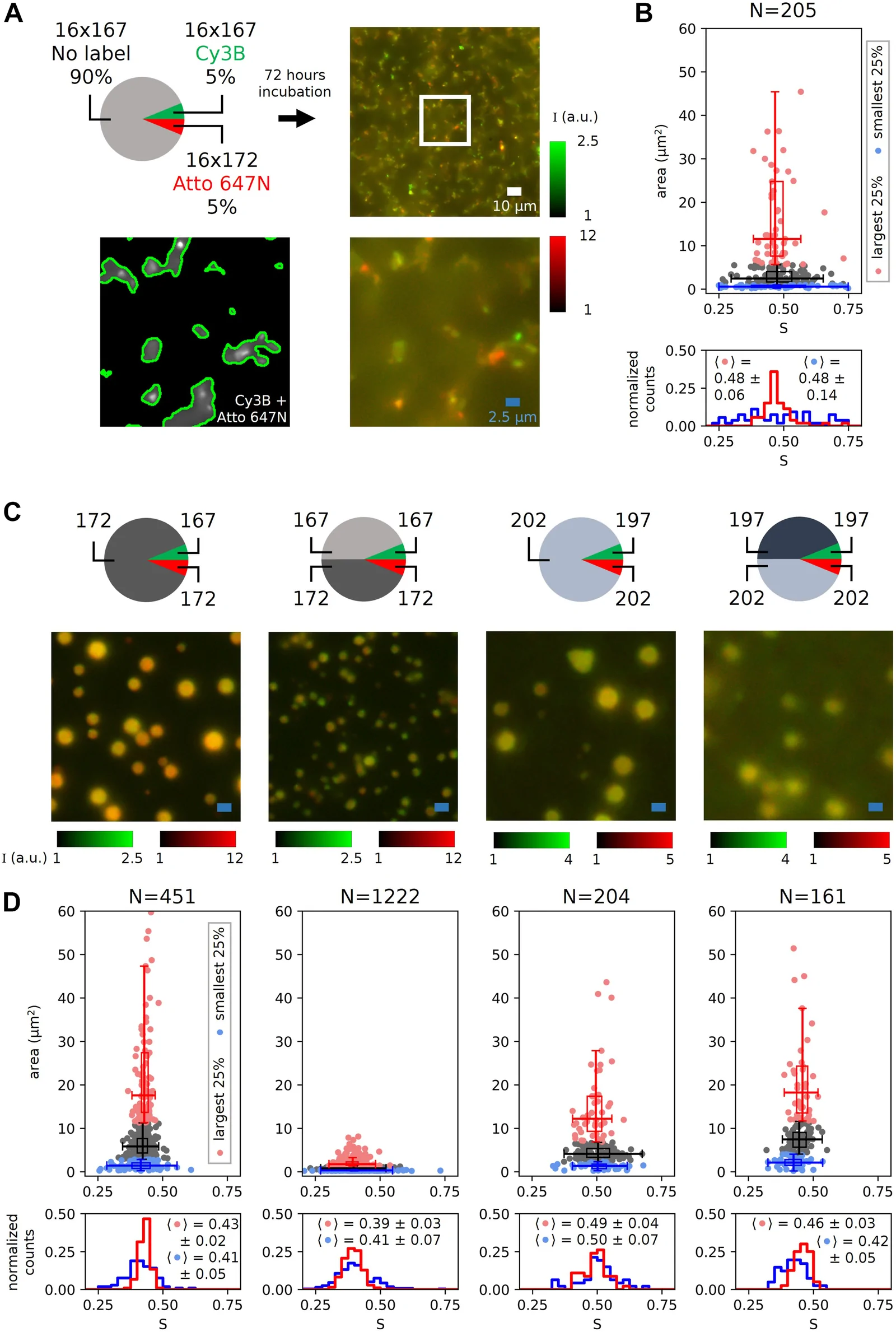
**Mixing fibers with different nucleosome repeat lengths leads to phase-separation of condensates with heterogeneous stoichiometries.***A*, *top left* - schematic of an NRL mixing experiment: 90% unlabeled 167 NRL fibers (*gray*) was mixed with 5% Cy3B-labeled 167 NRL fibers (*green*) and 5% Atto 647N-labeled 172 NRL fibers (*red*). *Right* - the resulting condensates. *Bottom left* - example of edge detection analysis of image. *B*, *top* - distribution of size, by area, of the largest 25% (*red*), smallest 25% (*blue*), and intermediate (*black*) condensates. *Bottom* - the largest 25% (*red*) and smallest 25% (*blue*) condensates show similar stoichiometry distributions. *C*, heterogeneous mixing of 167/172 and 197/202 NRL fibers. Increasing the 167–172 NRL fiber ratio from 1:19–1:1 leads to smaller condensates. *D*, size and stoichiometry distributions as in *B*. N is the number of analyzed condensates. Averages ⟨⟩ are shown as the mean ± SD. The scale bars represent 10 μm (*white*) and 2.5 μm (*blue*). Box plot whiskers represent 1.5 × IQR. NRL, nucleosome repeat length.

Having established how the phase-separation behavior of chromatin fibers is affected by linker lengths, we investigated the interaction between different NRL fibers. We made mixtures 167/172 NRL and 197/202 NRL chromatin fibers at varying stoichiometries. We labeled them with distinctive fluorescent dyes at the end of the DNA substrate (Fig. 3*A*, top left). To keep the ratio of the fluorescence intensity equal, the majority of the fibers was not labeled. For 95% 167 NRL and 5% 172 NRL chromatin we observed similar solid-like aggregates formation as for 100% 167 NRL (Fig. 3*A*, right). In addition, some clusters of exclusively 167 or 172 NRL fibers formed. To quantify the asymmetric distribution of fibers, we calculated the mean label stoichiometry $S=I_{Cy3B}/\left(I_{Cy3B}+I_{Atto647N}\right)$ (Fig. 3*B*). We did not observe a relation between the size of the aggregate and its stoichiometry. Smaller aggregates featured a wide dispersion of stoichiometries, which reflects the small number of fluorescent fibers within each condensate. We estimated that the smallest 167 NRL condensates only include one or no label (Fig. S8*E*). The considerable dispersion in S of the largest aggregates, on the other hand (red, top right histogram), suggests that condensates originally form as homogeneous clusters of fibers with the same NRL, but their selectivity for the same NRL decreases as they grow, and it becomes more likely to incorporate chromatin fibers of the other NRL. The excess of 167 NRL fibers still defines the morphology of the aggregates that resemble those of homogeneous 167 NRL (Fig. S4*A*).

For other mixtures of NRLs, we found circular condensates of various sizes. Condensates containing 95% 172 NRL and 5% 167 NRL chromatin (Fig. 3, *C* and *D*, left) were larger than those made of an equal ratio of 167/172 NRL fibers (Fig. 3, *C* and *D*, center left). This could be because increasing amounts of 167 NRL chromatin impedes the growth or merging of spherical condensates. Samples with 95% 202 NRL and 5% 197 NRL chromatin (Fig. 3, *C* and *D*, center right) and equal ratio 197/202 NRL (Fig. 3, *C* and *D*, right) showed similar morphology to the condensates formed by homogeneous 202 NRL chromatin. There was no difference in stoichiometry between large and small condensates (Fig. 3*D*, center right and right). Samples with 95% 197 NRL and 5% 202 NRL chromatin did not show condensate formation (Fig. S7), probably due to 197 NRL fibers being the least prone to condensate formation (Fig. S4*C*). These results show that chromatin fibers of different NRLs can be incorporated into the same condensate if mixed before inducing phase separation. The morphology of the resulting condensate is hardly affected by the minority of different NRL fibers. 167 NRL fibers are readily incorporated into 172 NRL fiber condensates, but reduce the size of the condensates when their stoichiometry is equal.

### Addition of different NRL fibers leads to domains within the condensate

**Figure 4:**
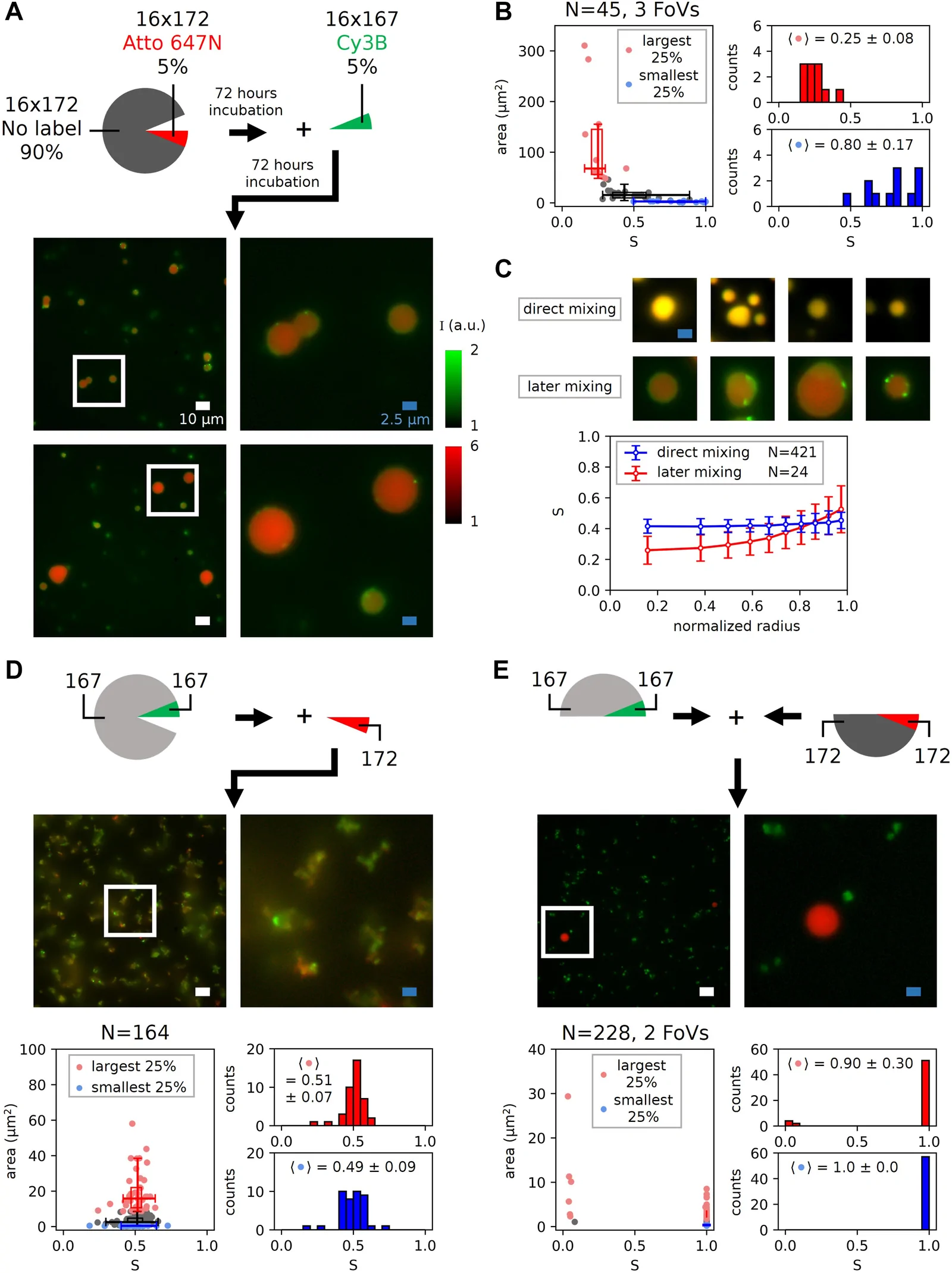
**Addition of 167 and 172 nucleosome repeat length fibers to previously formed condensates leads to incomplete incorporation.***A*, schematic of an NRL mixing experiment after initial phase separation. Chromatin phase-separation was induced with 90% unlabeled 172 NRL fibers (*gray*) and 5% Atto 647N-labeled fibers 172 NRL fibers (*red*). Cy3B-labeled labeled 167 NRL fibers (5%, *green*) were added after 72 h. Fluorescence microscopy 72 h after mixing of 172 NRL fiber condensates (*red*) with 167 NRL fibers (*green*) is shown below. *B*, *Left* - distribution of size, by area, of the largest 25% (*red*), smallest 25% (*blue*), and intermediate (*black*) condensates. *Right* - distribution of stoichiometries of the largest 25% (*red*, *top*) and smallest 25% (*blue*, *bottom*) condensates. The largest condensates contained mostly 172 NRL fibers and the smallest contained mostly 167 NRL fibers. *C*, comparison between direct and delayed mixing of 167/172 NRL fibers. *Top* - examples of condensates for the two mixing types. In both cases, 90% of the material was unlabeled 172 NRL fibers, 5% was Atto 647N-labeled 172 NRL fibers (*red*) and 5% was Cy3B-labeled 167 NRL fibers (*green*). *Bottom* - graph of label stoichiometries (S) as a function of the normalized distance to the detected center of mass of the condensate. When 167 and 172 NRL fibers were directly mixed, they were incorporated homogeneously (*blue*, data from Fig. 3). A total of 167 NRL fibers accumulate at the periphery with later mixing (*red*). Only condensates with an area larger than 10 pixels (1.3 μm^2^) were included in the analysis. Data points are mean ± SD. Lines connecting data points are to guide the eye and do not represent a fit of the data. *D*, similar to *A*, except 167 (90%) + 167-Cy3B (5%) NRL condensates were mixed with 172-Atto 647N (5%) NRL fibers. 172 NRL fibers were incorporated readily into the solid-like condensates formed by 167 NRL fibers. *E*, as in *A*, except 167 (45%) + 167-Cy3B (5%) NRL condensates were mixed with 172 (45%) + 172-Atto 647N (5%) NRL condensates. They did not mix. N is the number of analyzed condensates. Averages ⟨⟩ are shown as the mean ± SD. The scale bars represent 10 μm (*white*) and 2.5 μm (*blue*). Box plot whiskers represent 1.5 × IQR. IQR, interquartile range; NRL, nucleosome repeat length.

We next investigated the incorporation of different NRL fibers into condensates. Interestingly, when 167 NRL fibers were added to existing 172 NRL condensates, the 167 NRL fibers did not fully mix into the condensate, but rather accumulated at its surface (Fig. 4*A*). Incorporation of 167 NRL fibers clearly favored smaller condensates (Fig. 4*B*), most likely due to a larger surface to volume ratio. When the local label stoichiometry is plotted as a function of the distance to the condensate center, it is clear that 167 NRL fibers are less abundant in the core (Fig. 4*C*). Instead, the 167 NRL fibers that were added later clustered at the condensate periphery. This leads to an increase in S as the radius increases, confirming their presence almost exclusively at the edge of the condensate. While the 167 NRL fibers are localized primarily at the condensate periphery, their distribution along that surface is not homogeneous, as indicated by the bright spots along the condensate edge. This suggests a smaller-scale aggregation of 167 NRL fibers before they are merged with the larger 172 NRL condensate. In contrast, for condensates that formed with premixed 167/172 NRL fibers (5% 167 NRL, 95% 172 NRL), the condensate composition was uniform.

When the existing 167 NRL condensates were supplemented with 172 NRL fibers, the latter were readily incorporated (Fig. 4*D*, top). This incorporation was not condensate size-dependent (Fig. 4*D*, bottom). When preformed condensates of 167 and 172 NRL fibers were mixed after inducing phase separation, they did not seem to merge at all (Fig. 4*E*). The smaller size of the 167 NRL condensates in Figure 4*E* compared to those in Figure 4*D* is most likely due to the reduced concentration of 167 NRL fibers. Thus, existing spherical chromatin condensates can incorporate chromatin with different linker lengths, as long as those chromatin fibers have not yet formed condensates themselves. Interestingly, the addition of new fibers does not adhere to Ostwald ripening. Instead, it occurs through a multiphase condensate mechanism, involving a complex interplay between liquid-like and solid-like condensate properties. When chromatin is already present in both spherical and amorphous aggregates, they do not mix. The nature of preexisting condensates can therefore drastically influence the aggregation behavior of free fibers in solution and their merging behavior with newly formed condensates.

**Figure 5:**
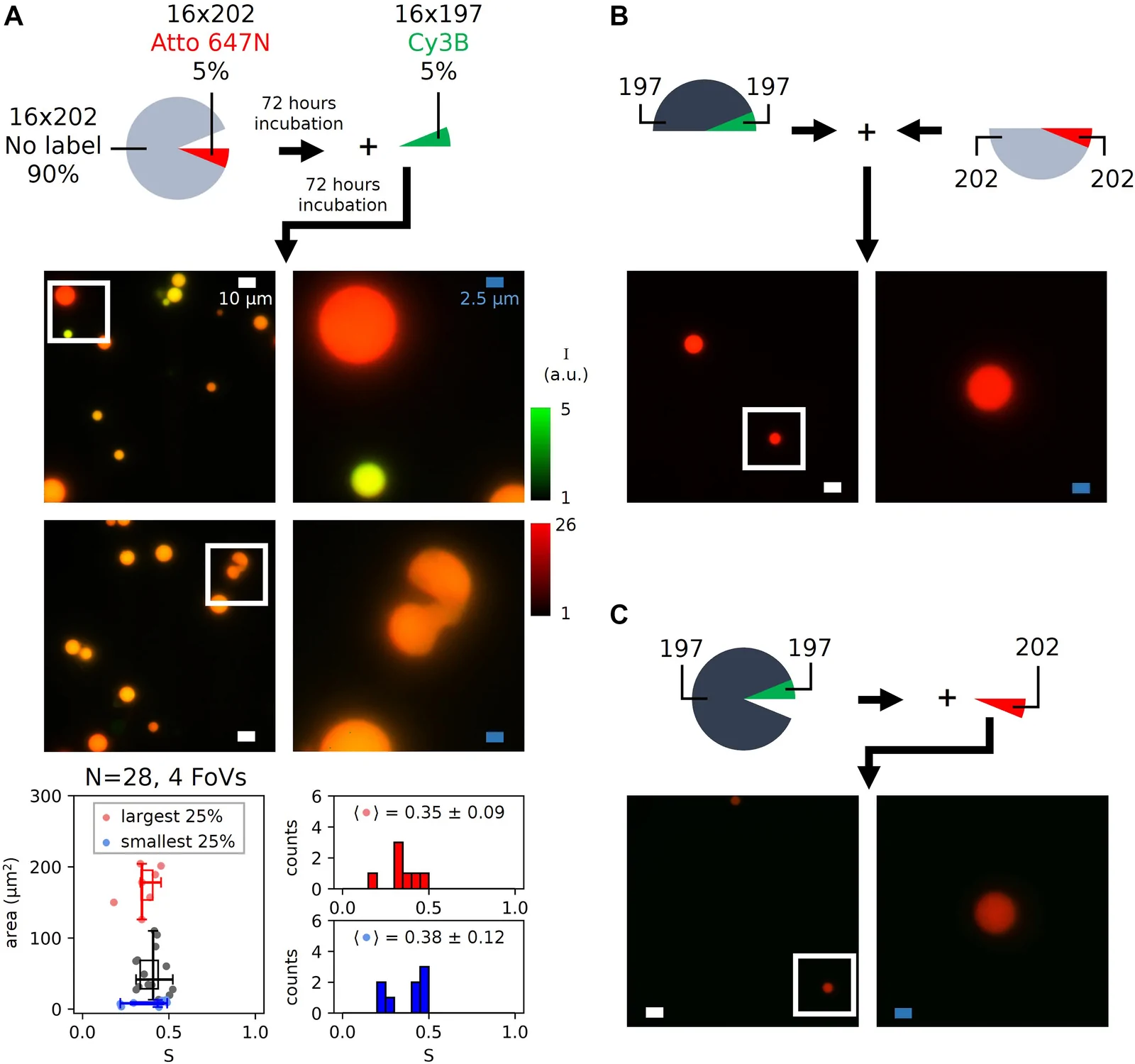
**197 NRL fibers are readily incorporated into existing 202 NRL condensates.***A*, *top*: schematic of an NRL mixing experiment after initial phase separation, as in Figure 4. *Center*: fluorescence microscopy of 202 NRL fiber condensates mixed with 197 NRL fibers. *Bottom left*: The size, by area, of the largest 25% (*red*), smallest 25% (*blue*), and intermediate (*black*) condensates did not affect the resulting stoichiometry. Although the stoichiometry varied between condensates, it was homogeneous within each, indicating liquid-like behavior when the condensate was formed and suggesting few merger events. Box plot whiskers represent 1.5 × IQR. *Bottom right* - distribution of stoichiometries of the largest 25% (*red*, *top*) and smallest 25% (*blue*, *bottom*) condensates. *B*, mixing of of 197 (45%) + 197-Cy3B (5%) NRL fibers with of 202 (45%) + 202-Atto 647N (5%) NRL fibers. *C*, mixing of 197 (90%) + 197-Cy3B (5%) NRL fibers after induction of phase seperation with 202-Atto 647N (5%) NRL fibers. N is the number of analyzed condensates. Averages ⟨⟩ are shown as the mean ± SD. The scale bars represent 10 μm (*white*) and 2.5 μm (*blue*). NRL, nucleosome repeat length; IQR, interquartile range.

When samples containing 202 NRL were complemented with 197 NRL fibers, the morphology of the condensates (Fig. 5*A*) resembled that of 202 NRL-only condensates (Fig. 2*A*, fourth row). The 197 NRL fibers were incorporated into these condensates, as is evident from the shift in average S from ∼ 0 to 0.38 ± 0.09 (mean ± SD), independent of the condensate size (Fig. 5*A*, bottom). Notably, the aggregates exhibited strikingly different colors after mixing. Since we estimated that thousands of 202 NRL fibers were included in a single condensate (Fig. S8*E*), statistical variations in the number of labeled fibers cannot explain the uneven distribution in S. It is, therefore. more likely that the 197 NRL fibers formed small aggregates before merging with the existing condensate. In that case, the statistical variation comes from a small number of mergers, rather than a small number of fibers that were incorporated. For delayed mixing of equal ratio 197 NRL fibers with 202 NRL fibers (Fig. 5*B*), only N = 5 condensates were observed in six fields of view. The few condensates that were present were almost exclusively comprised of 202 NRL fibers. Similarly, for majority 197 NRL fibers only N = 2 aggregates were found (Fig. 5*C*), confirming that at these concentrations the 197 NRL fibers hardly form condensates.

## Discussion

Many parameters determine the phase-separation behavior of chromatin. Important factors are mono- and divalent salts (35, 43, 54, 55, 63), crowding agents (54, 55, 56), nucleosome repeat number (43, 53, 55, 64), NRL (43, 54), histone modifications (43, 53, 65, 66, 67, 68), histone variants (69), linker histones (43, 70, 71), or the presence of other DNA- or histone-binding proteins (67, 72, 73, 74, 75). Here we focused on the internal conformation of chromatin fibers as defined by their linker lengths.

In this work, we have shown fundamental differences in chromatin phase separation behavior between homogeneous and heterogeneous mixtures of different NRL fibers. To obtain sufficient amounts of (fluorescently labeled) substrates, we introduced a novel method for producing large quantities of nucleosome arrays with varying NRLs without the need for gel purification. In homogeneous NRL mixtures of this material, 172 and 202 NRL fibers formed spherical condensates, while 167 and 197 NRL fibers formed amorphous aggregates. When fibers of different NRLs were mixed before inducing phase separation, the final condensate morphology was determined primarily by the most abundant NRL. The incorporation of sparse 172 NRL fibers into 167 NRL condensates was more homogeneous than the incorporation of sparse 167 NRL fibers into 172 NRL condensates. In the latter case, small clusters of 167 NRL fibers formed at the periphery of the already formed 172 NRL aggregates. Furthermore, 167 NRL fibers were absorbed from solution, but then did not mix inside the condensate. Instead, they formed an outer shell as a multiphase condensate not observed with other NRLs.

This morphology appears similar to the perinucleolar chromatin surrounding the nucleolus (76, 77), and more generally to the division between condensed heterochromatin and sparser euchromatin regions. Since shorter NRLs are typically associated with euchromatin and longer NRLs with heterochromatin (78), the cell could preferentially localize and compact specific parts of its genome by controlling the NRL, which, as we show here, affects the mixing and coalescence behavior of chromatin condensates. This process suggests an equilibrium between self-association of fibers with identical NRLs and the incorporation of these fibers into larger condensates. Phase separation is then not just a tool used by the cell to locally concentrate genomic material, but also to selectively partition sequences based on their nucleosome spacing. This model could be further expanded to describe the intrinsically sequence-dependent organization of the genome: DNA sequence affects nucleosome positioning (79, 80), thereby influencing linker length, which in turn governs local nucleosome stacking, chromatin compaction, and ultimately higher-order compartmentalization through phase separation.

An as of yet unanswered question is how cells establish and maintain the boundaries between different chromatin domains (*e.g.*, euchromatin and heterochromatin) *in vivo*. The formation of 167 NRL fiber subdomains localized at the surface of 172 NRL condensates (Fig. 4) suggests a novel physical mechanism by which boundaries between topologically distinct regions of the genome can be controlled, without the continuous presence of additional chromatin structuring proteins. Chromatin remodelers might instead stabilize the condensate shell to potentially regulate transcription factor or RNA polymerase access to the DNA core by modifying its NRL. Indeed, Chen *et al.* (81) have shown that the ISWI remodeler can regulate condensate formation *in vitro* by altering fiber NRL. Depletion or overexpression of specific chromatin remodelers could therefore probe how NRL affects condensate development *in vivo*, although targeting specific loci may be challenging. It would be particularly interesting to see whether chromatin fibers are selectively processed by NRL within mixed condensates and how this influences dynamics of shells composed of amorphous chromatin, as this could offer a potential route through which these condensates can be resolved and their contents made accessible again. Such mechanisms could be verified by detailed colocalization microscopy and multiscale modeling of these complex condensates.

The difference in phase-separation behavior of chromatin with different linker lengths was first reported by Gibson *et al.* (43). They proposed a binary model in which chromatin with 10n + 5 bp of linker DNA phase separates, whereas 10n bp of linker DNA does not. Moreover, in the latter case, phase separation could still be induced with sufficient Mg(OAc)_2_. The 10-bp periodicity of the linker DNA determines the relative orientation of neighboring nucleosomes and thus mediates the strength of histone stacking interactions. They suggested that this would be a driving factor in 30 nm fiber formation (5, 82). Chromatin with 10n bp linker favors local compaction through cis nucleosome stacking mediated by interactions of the H4 tail with the acidic patch of an H2A/H2B dimer of a neighboring nucleosome from the same fiber. In addition, linker histones can alter the relative orientation of linker DNA and nucleosome, and stabilize cis interaction (83, 84). Compaction at a larger scale may require weaker trans interactions of the same H4 tails, but with H2A/H2B acidic patches from other, distant chromatin fibers. However, these may be unavailable due to existing cis-stacking interactions. Conversely, looser ’beads on a string’ 10 nm fibers with 10n + 5 bp linkers, while less compact at the scale of a single chromatin fiber, would be compacted more easily through trans supramolecular interactions into phase-separated aggregates. Chen *et al.* (81) reported a gradual shift in nucleosome stacking preference from inter- (*trans*) to intra- (*cis*) molecular interactions for fibers with linker lengths ranging from 25 to 30 bp, conforming to the 10n and 10n + 5 bp linker dependence of nucleosome-nucleosome interaction preferences leading to fiber-fiber interactions or phase separation. Here, we expanded this model and observed the consequences for binary mixtures of different NRL fibers.

There is, however, a fundamental difference in the topology of the higher-order folding structure between 167 and 197 NRL fibers (both 10n linker) (85, 86). From structural studies, it is known that 167 NRL fibers feature a two-start topology, while 197 NRL fibers are far less ordered and force spectroscopy has revealed a one-start topology (57, 87). As a consequence, 167 NRL folded fibers are folded more robustly, which further limits their ability to unstack and allow for trans-stacking interactions. Indeed, we observed significant differences between both NRLs, showing a propensity for larger fibrous, solid-like aggregates in the 167 NRL samples.

While neighboring nucleosome orientation, directed by the size of the 10n or 10n + 5 bp linker, is a good predictor of local chromatin structure, it may be better represented by an equilibrium of various degrees of nucleosome stacking in cis. This equilibrium critically depends on the linker DNA bending energies, nucleosomal DNA unwrapping energies, histone stacking energies and stacking topology (*i.e.* which nucleosomes in the fiber are stacked with each other). In this sense, we interpret the long and fibrous aggregates formed by the 167 NRL fibers as multimers of internally stacked nucleosomes. In these fibers, stacking into a 2-start configuration is favored by short, inflexible linker DNA (28, 37). This organization only leaves the nucleosomes at the start and end of the two nucleosome columns available to interact with other fibers, giving the resulting aggregates a directionality along the fiber’s major axis. Force spectroscopy measurements of 197 NRL fibers showed reduced nucleosome stacking stability compared to 167 NRL fibers (86). This structure exposes half the nucleosomes to interactions with other fibers (one per end) relative to the two-start configuration (two per end). This may explain the reduced tendency for condensate formation.

Strickfaden *et al.* (56) reported LLPS only under particular conditions. The presence of DTT, BSA, and acetate were all required. We only included the first two in our experiments. The solid-like fibrous aggregates formed by 167 NRL chromatin most resemble the morphology of the chromatin condensates formed in C3H/10T1/2 mouse cells, shown in the same paper. However, longer (∼ 40 bp) linker lengths are typically more abundant in mouse embryonic cells (88) and it is difficult to extrapolate our results to the *in vivo* situation.

Later work by Gibson *et al.* (55), in an attempt to reconcile the differences between these and their previous results, showed liquid-like properties even without DTT or BSA through extensive FRAP and condensate fusion experiments. The difference in DTT and BSA-dependent behavior was suggested to be a consequence of their ability to inhibit photo-crosslinking of chromatin condensates by radical oxygen species produced during laser excitation, thus preserving mobility. The exact mechanism by which DTT and BSA achieve this, however, remains unclear. In addition, they showed solid-like behavior at 4 mM Mg(OAc)_2_, which is consistent with the results of our FRAP measurements that were likewise performed under high-magnesium conditions.

Interestingly, Chen *et al.* (54) reported LLPS for 15 × 197 NRL fibers, while 62 × 202 NRL fibers showed solid-like aggregation behavior. Their results also showed that the addition of BSA leads to a shift towards more solid-like fibrous structures at the magnesium concentration we measured (10 mM), as reported in (56). Their 202 NRL experiments did not contain BSA, unlike the experiments reported in this study. Their data also suggested that longer fiber lengths inhibited LLPS. Considering the length of chromatin in topologically active domains (100–1000 kbp) (19, 89, 90), the relevance of experiments with substrates of <100 kbp can be questioned. Most likely, there are further chromatin-binding and -bridging proteins that greatly facilitate the phase separation of native chromatin (50, 91). In that regard, the use of our purified system, which enforces defined NRLs without nonhistone proteins, does have inherent limits. While it has enabled us to capture the effects of different chromatin fiber organizations on the formation of condensates it does not capture the irregularity of nucleosome positions of chromatin *in vivo*, which may moderate the effects observed here. This, in combination with the factors outlined at the start of this section, could further modulate NRL-driven condensate formation.

The timescale of our measurements is also worth considering. Since we observed condensates over a micron in size, the initial spinodal decomposition and nucleation processes have, for the most part, been completed (92). Our FRAP measurements revealed solid-like properties for condensates of all measured NRLs, which we attribute to the system being in a metastable, kinetically trapped state, most likely driven by the dominance of strong stacking interactions under high magnesium concentrations (93). These findings, in combination with the observation that 167 NRL fibers can form a shell over 172 NRL condensates, suggest a potential regulatory mechanism in which chromatin boundaries are fine-tuned by condensate rigidity and permeability, rather than by local chromatin fiber configuration. These parameters could be probed by atomic force microscopy and fluorescence microscopy respectively. The exact mechanisms underlying the condensate aging process in chromatin condensates, however, remain poorly understood (94) and warrant further investigation.

Our experiments suggest an interesting mechanism with which cells can robustly control the formation of chromatin aggregates, and directly link this process to the genetic content: by controlling the linker length, either by the DNA sequence that positions the nucleosomes, or by chromatin remodelers that reposition nucleosomes, the cell may control the level of chromatin aggregations, and so indirectly control gene activity. One can even envision that evolutionary selection may have contributed to such control. However, *in vivo* tests will need to be conducted to test the robustness of the reported sequence dependence.

## Experimental procedures

### Construction of plasmids containing 601 arrays

To obtain the DNA substrates used for aggregation experiments, pUC18 plasmids containing an array of 16 Widom 601 nucleosome positioning sequences (44) with varying linker lengths were created as described in Brouwer *et al.* (86), using a method developed by Wu *et al.* (45). To prepare for later Golden Gate assembly, BsaI restriction sites were introduced between the array and the plasmid backbone by cloning two sets of oligos at unique restriction sites on both 3′ → 5′ ends (supplementary materials). 6 μM of both oligos were annealed in an annealing buffer (10 mM Tris-HCl pH 8, 1 mM EDTA, and 50 mM NaCl) at 95 C for 2 min. The plasmid containing the 601 array was digested at the EcoRI and SapI sites. 10 μg plasmid DNA was digested with 20 U EcorI (NEB R0101) and 10 U SapI (NEB R0569) for 1 h at 37 °C in a CutSmart buffer (NEB B7204). The linearized fragment was purified from a 1% agarose, 1× TB gel using a gel extraction kit (QIAgen 28704: QIAquick Gel Extraction Kit). The fragment was then inserted by mixing 130 ng of the plasmid, 13 ng of the insert, and 2000 U T4 ligase in a T4 ligase buffer (NEB B0202) at 16 °C overnight (>16 h). Following T4 deactivation at 65 °C for 10 min, 20 ng of the plasmid was used for transformation of E.coli XL-1 Blue competent cells by heat shock for 2 min at 42 °C. LB medium was added to the cells, and they were then gently shaken (240 rpm) at 37 °C for 30 min before being spread on LB agar plates with added 100 μg/ml ampicillin. Cultures were grown overnight. The amplified plasmid DNA was harvested with a midiprep kit (Thermo Fisher Scientific K0481: GeneJET Plasmid Midiprep Kit) according to the manufacturer’s instructions. These steps were repeated for the opposite side of the backbone with XbaI (NEB R0145) and NdeI (NEB R0111) to introduce a BsaI restriction site on the other side of the plasmid. A third BsaI site was natively present in the pUC18 plasmid backbone.

### Production of labeled DNA substrates

Hairpins compatible with BsaI sites were annealed in the annealing buffer. The sequences can be found in the supplementary materials. A fraction of the hairpins was made from fluorescently labeled oligos with the label attached to a modified base in the hairpin. The hairpins were ligated to the ends of the 601 array by Golden Gate assembly. An 8:1 M ratio of hairpin to plasmid vector was used, with 20 to 200 μg plasmid DNA. A ratio of 5 μg DNA: 1 U BsaI-HFv2 (NEB R3733) and 5 μg DNA: 100 U T4 ligase was used, in a CutSmart buffer with 1 mM ATP added. The assembly protocol consisted of 20 cycles of 15 min at 37 °C then 5 min at 16 °C. T5 exonuclease (NEB M0663) was then added with a ratio of 4 μg DNA: 1 U T5 to remove the backbone. The hairpin-capped 601 array was purified to remove the T5 and free nucleotides (Promega A9281: Wizard SV Gel and PCR Clean-Up System). A comprehensive overview of the resulting substrates can be found in Figure S2, with their yields in Table S1.

### Chromatin reconstitution

Chromatin was reconstituted through salt gradient dialysis, based on the method described in Kaczmarczyk *et al.* (95). Further validation of this method can be found in (87, 96, 97). 601 arrays were mixed with recombinant human histone octamers (EpiCypher 16-0001: Histone Octamer, Recombinant Human) in three stoichiometric ratios between 0.7:1 and 1.1:1 DNA:octamer in a 2 M NaCl, 1× TE buffer, pH 7.5.

A total of 5 μg of DNA was reconstituted at a final concentration of 10 nM. In addition, 250 ng competitor DNA was added. Samples were pipetted into 10 kDa molecular weight cut-off low-binding membrane cups (Thermo Fisher Scientific 69572: Slide-A-Lyzer MINI Dialysis Devices, 10K molecular weight cut-off) and placed in 200 ml of a 2 M NaCl, 1× TE pH 7.5 (high salt buffer).

Samples were reconstituted overnight through salt gradient dialysis at 4 °C. A peristaltic pump was used to flow in 1 L of a 1× TE pH 7.5, 10 mM NaCl buffer at 0.9 ml/min into the high salt buffer, while pumping out at an equal volume after mixing.

To assess the quality of the reconstitution, 4 μL of each reconstitution was loaded onto a 0.8% agarose, 1× TB gel and run for 90 min at 150 V. All samples were loaded with 10% glycerol. Reconstituted chromatin will have a reduced gel shift when compared to the DNA-only band, see Figure S1.

### Assessment of chromatin reconstitution efficiency

To determine the quality of the reconstituted chromatin, chromatin samples were digested at a ratio of 200 ng DNA: 1 U MfeI-HF (NEB R3589) for 1 h at 30 °C. The Widom 601 sequence contains a single MfeI recognition + cut-site 28 bp in. Fully reconstituted nucleosomes prevent MfeI digestion, while incompletely reconstituted chromatin fibers are cut. The size distribution of these fragments reflects the integrity of the assembled nucleosome arrays. Samples were then diluted to 1% SDS to remove all histone proteins from the DNA and deactivate MfeI. Samples were then placed on gel (Fig. S3). 700 bp competitor DNA without MfeI site was used as a control for specificity.

A total of 4 μL of each sample was loaded onto a 1.0% agarose, 1× TBE gel and run for 120 min at 150 V. The gel was stained postrun with Safe-Red (ABM G680).

### Phase separation

Reconstituted chromatin was diluted to 20 nM in 25 mM Tris-HCl pH 7.5, 5 mM DTT, 0.1 mM EDTA, 0.01% w/v BSA, and 1 mM Trolox and was incubated for 15 min. Phase separation was induced by adding a phase separation buffer, to a final concentration of 17 nM chromatin in 100 mM NaCl and 10 mM MgCl_2_ in low-binding tubes (Eppendorf 0030108132: Protein LoBind Tubes). Samples were incubated for 72 h at 4 °C before measurement.

To ensure integrity of the condensates and adherence to the glass-bottom 384-well plates (Greiner Bio-One 781892: Sensoplate 384-Well Glass-Bottom Plates) or glass-bottom 25-well plates (home-built), each well was cleaned, etched and coated. Wells were washed with 25 μL HPLC-grade water and subsequently cleaned with 10 μL HCl 5M for 30 min, followed by two more washing steps with 25 μL HPLC-grade water. Next, the wells were etched with 10 μL NaOH 5M for 30 min and then washed twice with 10 μL HPLC-grade water. The surface was then coated with 10 μL 2% w/v BSA overnight. Finally, the wells were washed twice with 10 μL HPLC-grade water.

A total of 20 μL chromatin per well was left to sediment. Wells were covered with tape to avoid evaporation during measurements.

For measurements performed 2 h after inducing phase separation (Fig. S5), rapid attachment of the condensates was required. The protocol is identical to the previous, except no HCl was used and BSA coating step was replaced by 0.1% w/v PLL (Sigma-Aldrich P8920) for 1 h.

### Fluorescence microscopy

Measurements were performed with a Nikon Eclipse Ti microscope with a 100×, NA 1.45, plan APO oil-immersion objective. Images were recorded with an Andor XU-888 camera operated by Nikon Imaging Software. Samples were excited with a Lumencor MultiLaser. Excitation wavelengths were 549 nm (Cy3B) and 632 nm (Atto 647N). Measurements (typically a few hours) were performed at RT. Each sample was scanned and one FoV (133.2 × 133.2 μm, 1024 × 1024 pixels) was captured if it was considered to be representative of the sample. If this was not possible, up to five more FoVs were recorded. Measurements used three reconstituted replicates of each NRL fiber (Cy3B, Atto 647N, and unlabeled).

### FRAP

FRAP measurements were performed with a Nikon Eclipse Ti microscope with either a 60 ×, NA 1.4, plan APO oil-immersion objective (Fig. S5) or 100×, NA 1.45, plan APO oil-immersion objective (Fig. S6), and Spinning Disk Confocal module. Images were recorded with a Prime 95B camera operated by Nikon Imaging Software. Samples were excited with a Lumencor MultiLaser. Excitation wavelength was 561 nm (Cy3B). Measurements (typically a few hours) were performed at RT. Each sample was scanned and several condensates within each FoV (112.6 × 112.6 μm, 1024 × 1024 pixels - Fig. S5; 69.3 × 99.0 μm, 770 × 1100 pixels - Fig. S6) were partially bleached. Frames were taken every second for 5 s before bleaching. Frames were taken every 5 s for 5 to 10 min after bleaching.

### Image analysis

Fluorescence microscopy images were analyzed with home-built python software that makes use of the OpenCV package (98). Images were denoised with a non-local means denoising algorithm (99) using an h=10 filtering parameter, a 7 pixel template window size and 21 pixel search window size. Condensates were segregated through binary thresholding of the combined Cy3B and Atto 647N images. Intensity thresholds were typically $0.4*I_{\max}$, with further adjustments ($\pm 0.1*I_{\max}$) done by eye. The circularity of each condensate was calculated as $4\pi A/P^{2}$, with A its area and P its perimeter. Objects with an area smaller than 10 pixels (0.17 μM^2^) were not included in the analysis. The ratio between Cy3B and Atto 647N signal intensity of a condensate was quantified from its stoichiometry $S=I_{Cy3B}/\left(I_{Cy3B}+I_{Atto647N}\right)$, with $I_{Cy3B}$ and $I_{Atto647N}$ the intensity of each pixel within detected condensates in the Cy3B and Atto 647N channel, respectively.

## Data availability

The fluorescence microscopy data used in this article are available in a public repository:

https://surfdrive.surf.nl/s/CTsCSyMbKjSQEJd

as ’DataErnstChromatin.zip’.

## Supporting information

This article contains supporting information.

## Conflict of interest

The authors declare that they have no conflicts of interest with the contents of this article.
