# Supplementary material for: Mixing chromatin fibers with different nucleosome repeat lengths changes dynamics of chromatin phase separation
Source: J Biol Chem. 2026 Aug 11;302(10):113386. doi: 10.1016/j.jbc.2026.113386 (PMC13599634; doi:10.1016/j.jbc.2026.113386)
Supplement: Supplementary file 1 [file mmc1.pdf]

Supporting Information

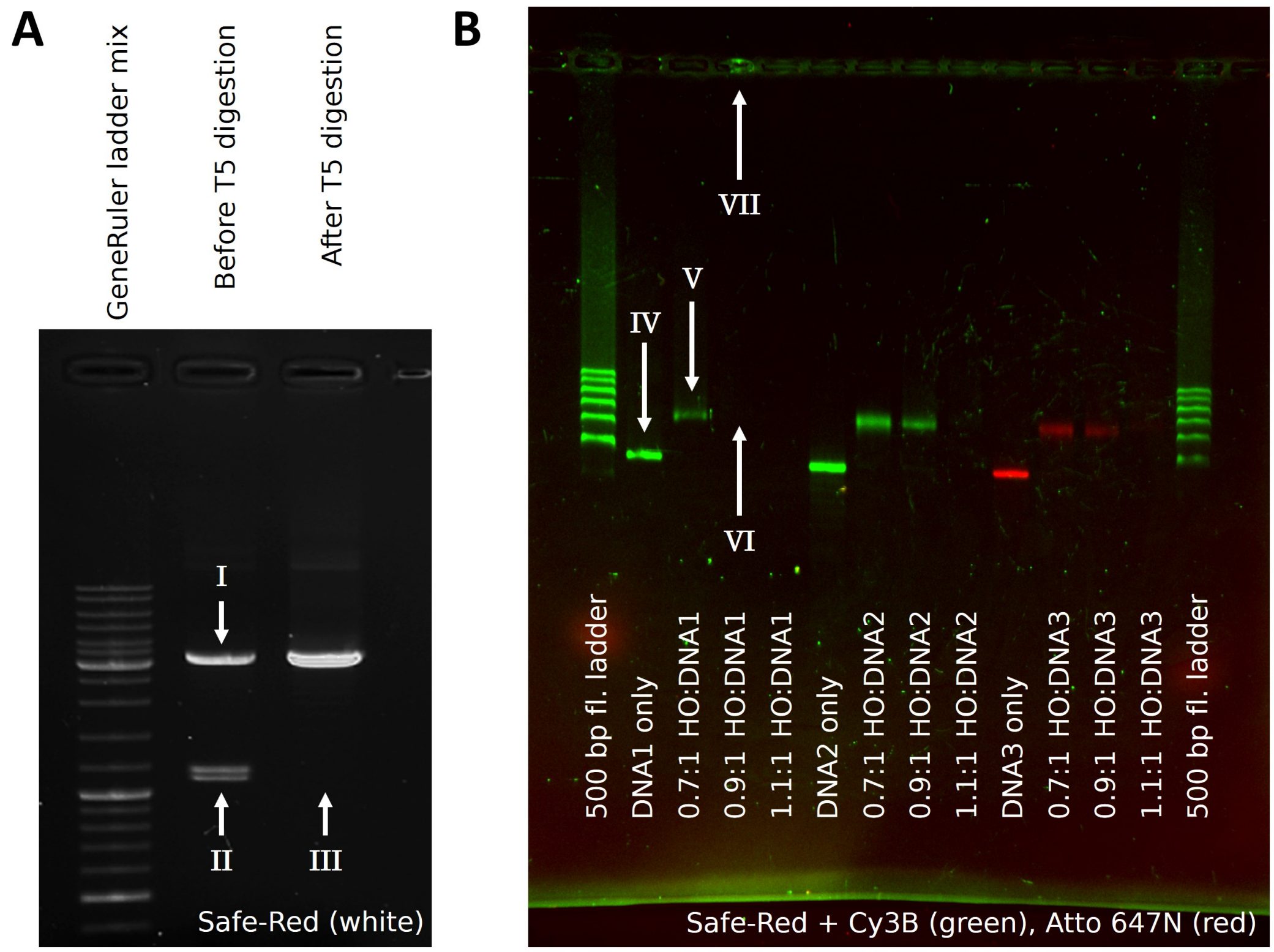

Figure S 1: **Golden Gate assembly and T5 exonuclease digestion produce pure chromatin fibers in large quantities without gel extraction.** Plasmids containing 16 repeats of the desired NRL were digested and ligated to hairpins. The remaining backbone was digested using T5 exonuclease. Chromatin was then reconstituted through salt gradient dialysis. **A)** Example agarose gel of digested 16 × 172 NRL plasmid with ligated hairpins (**I**) and plasmid backbone fragments (**II**). Uncapped backbone fragments were digested after T5 exonuclease treatment (**III**). Ladder is GeneRuler mix (ThermoFisher SM0331). **B)** Native agarose gel of salt gradient dialysis reconstitution. DNA1 contained unlabeled hairpins and was reconstituted at 50 nM, and stained with Safe-Red (ABM G680) for visualization on gel (**IV**). Reconstitutions were performed over multiple histone octamer (HO) to DNA ratios. Reconstituted chromatin shows reduced gel migration (**V**). Excessive HO : DNA ratios cause aggregation during reconstitution (**VI**), some of which can be seen in the slot (**VII**). DNA2 contained Cy3B-labeled hairpins and was reconstituted at 10 nM. DNA3 contained Atto 647N-labeled hairpins and was reconstituted at 10 nM. The Safe-Red and Cy3B fluorescence channel is shown in green and Atto 647N in red. Ladder is 500 bp fluorescent (Jena M-236S).

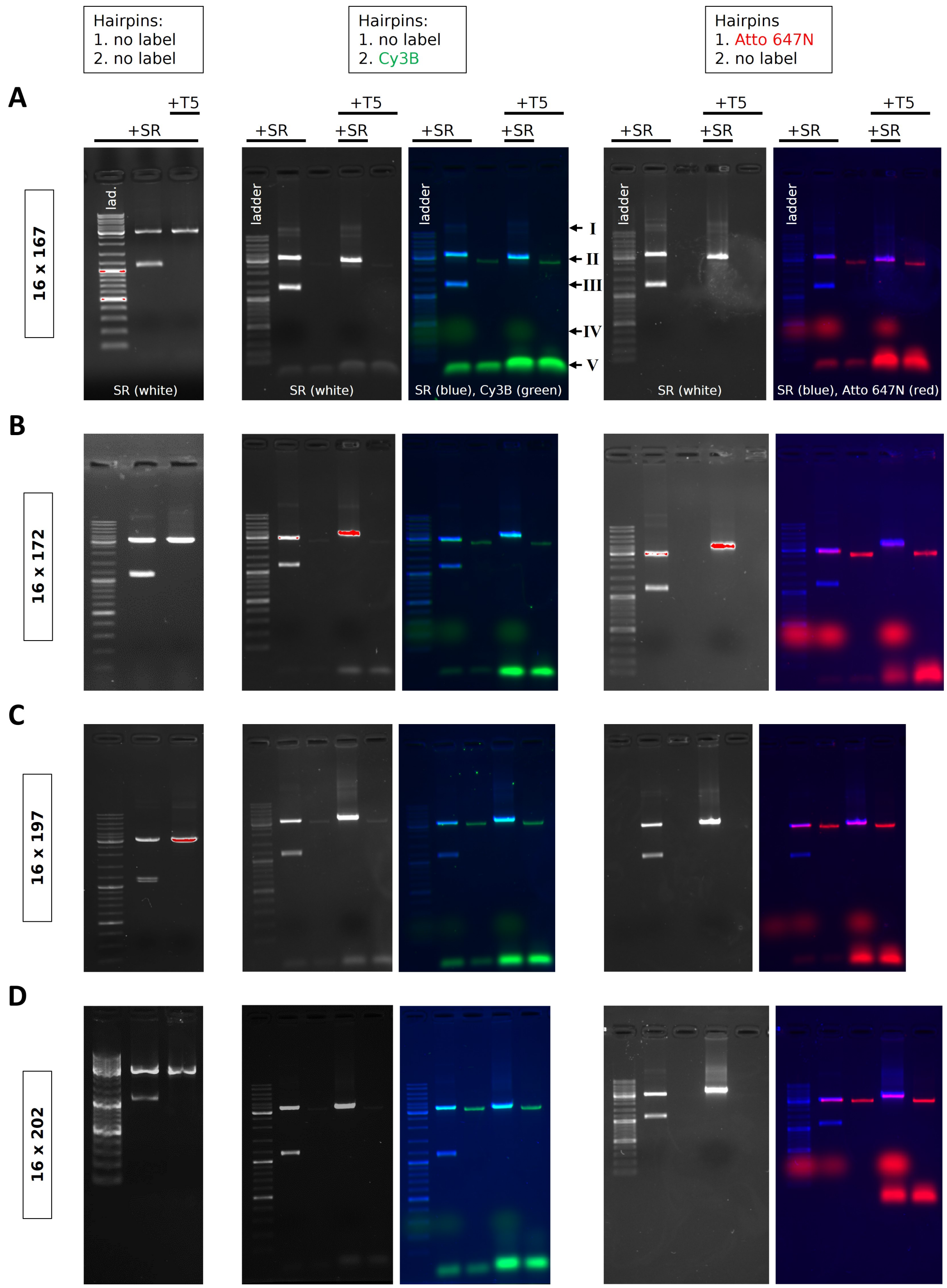

Figure S 2: **Overview of all DNA substrates created with hairpin ligation and T5 exonuclease.** Ladder: GeneRuler mix (ThermoFisher SM0331). SR: nucleic acid stain Safe-Red (ABM G680). +SR: sample in channel was loaded with Safe-Red loading dye. +T5: sample was incubated with T5 exonuclease, as described in the methods section. Red pixels in the SR (black/white) channels indicate signal over-saturation. **A)** 16 × 167 NRL substrates with no label (left), Cy3B-label (center two) and Atto 647N label (right two) on hairpin. (**I**) Trace amount of undigested plasmid. (**II**) 16 × 167 NRL array with ligated hairpins. Safe-Red loading dye causes a small shift in bands (+SR). (**III**) Band with both plasmid backbone fragments (1096 and 1147 bp) that will be removed by T5 exonuclease (+T5). (**IV**) Loading dye tracker. (**V**) Unligated hairpins with fluorescent labels. **B)** Same as in A), but for 172 NRL. **C)** Same as in A), but for 197 NRL. **D)** Same as in A), but for 202 NRL.

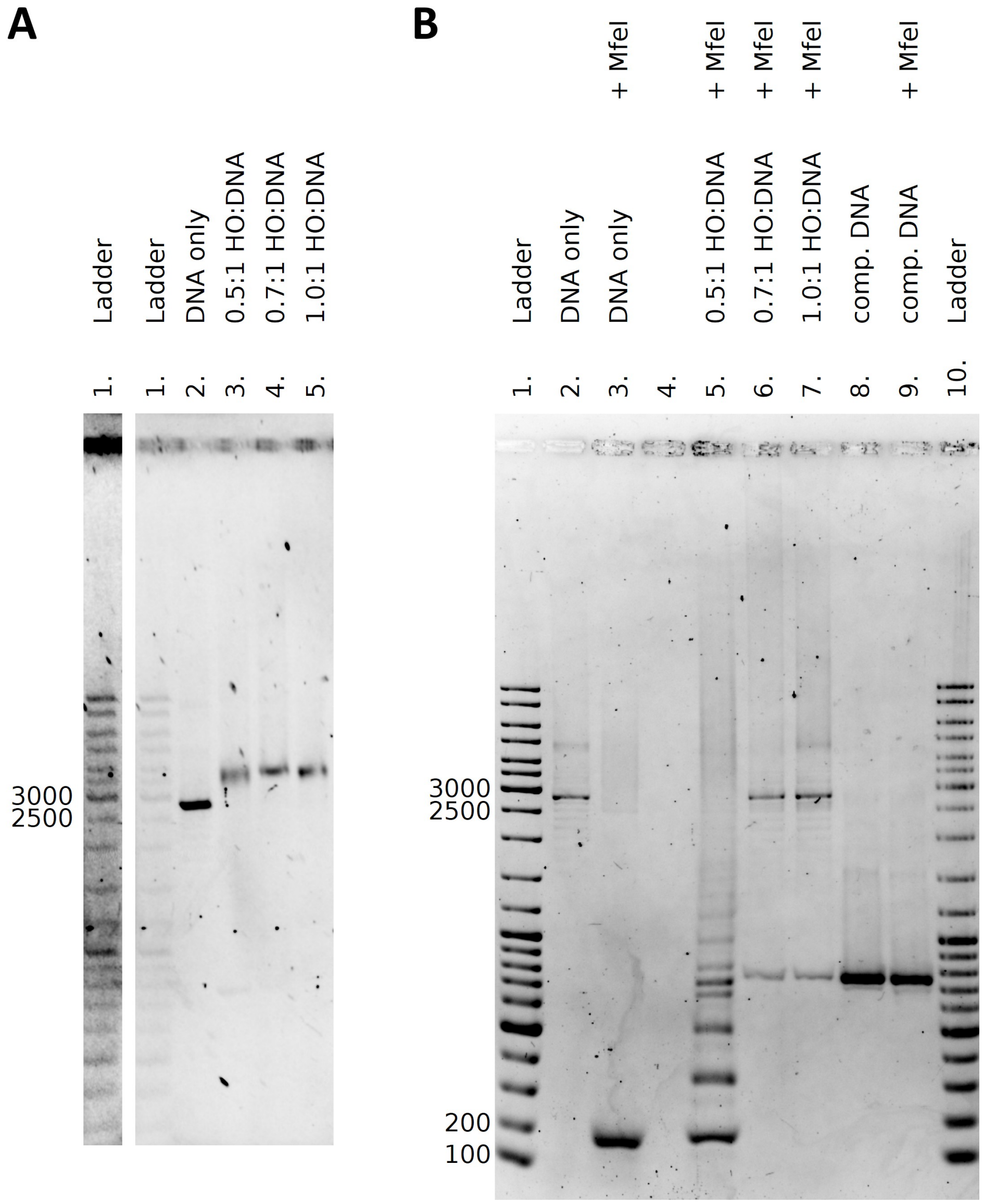

Figure S 3: **Assessment of chromatin reconstitution efficiency using MfeI digestion. A)** Native agarose gel of 16 × 172 NRL chromatin reconstitution. Ladders (1.) are the same lane, with increased contrast on the left. Histone octamer (HO) to DNA ratios were gradually increased to obtain optimal reconstitution, causing a gel-shift (3.- 5.) compared to the DNA only band (2.). Crisp, well defined bands indicate complete reconstitution. **B)** Quality control of the reconstituted chromatin using MfeI digestion. Unprotected DNA (2.) was digested into 172 bp fragments (3.) at the MfeI site on each 601 sequence. Partially reconstituted chromatin (5.) was digested where there were no nucleosomes to protect the MfeI site, yielding bands of multiple sizes. Fully reconstituted chromatin (6.-7.) had nucleosomes positioned on all 601 sequences so was not digested. Competitor DNA with no MfeI restriction sites (5.-9.) was added as a control for non-specific MfeI activity. Ladder: GeneRuler mix (ThermoFisher SM0331).

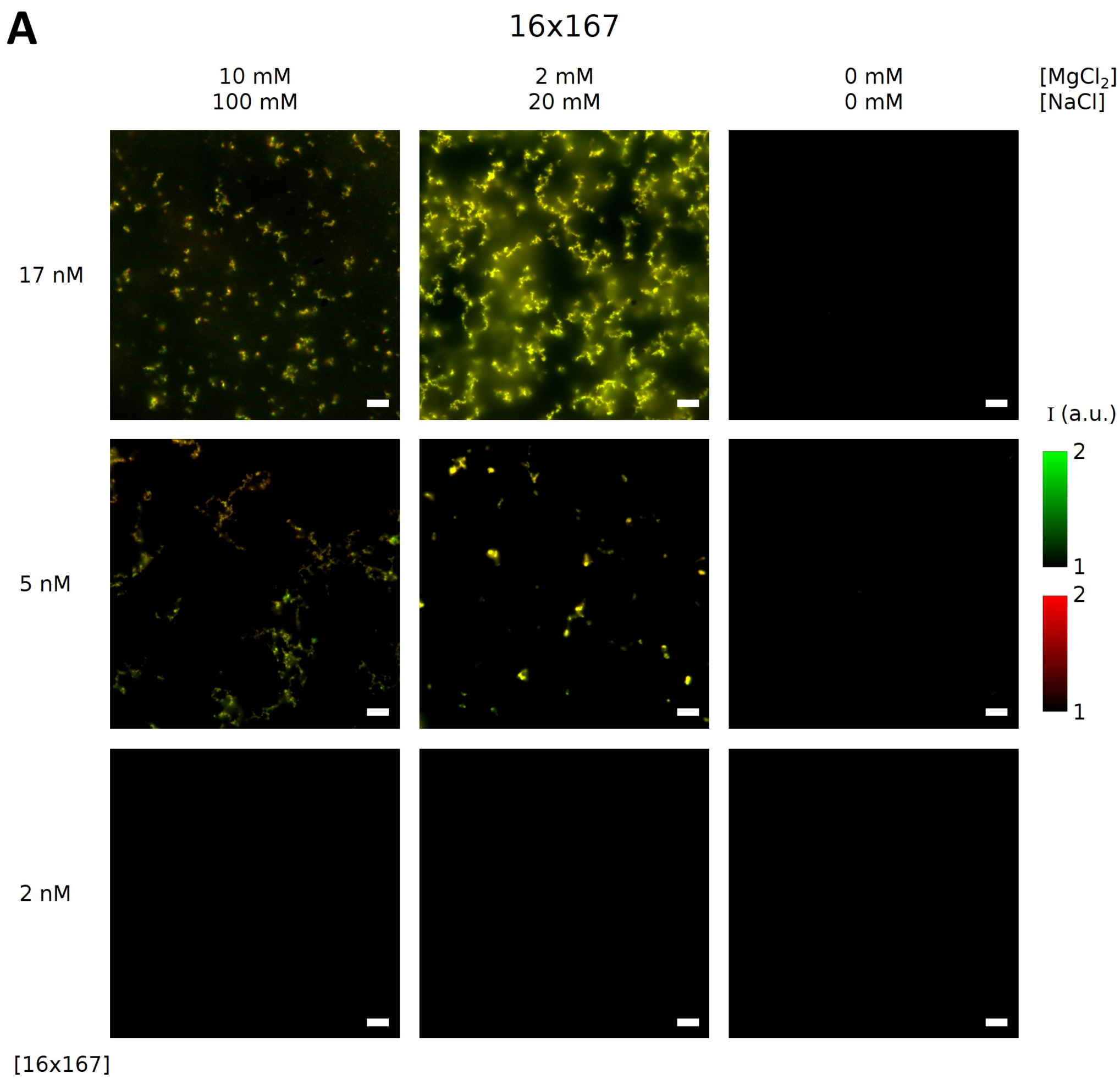
A
16x167
10 mM
100 mM
2 mM
20 mM
0 mM
0 mM
[MgCl2]
[NaCl]
17 nM
5 nM
2 nM
[16x167]
I (a.u.)
2
1
2
1

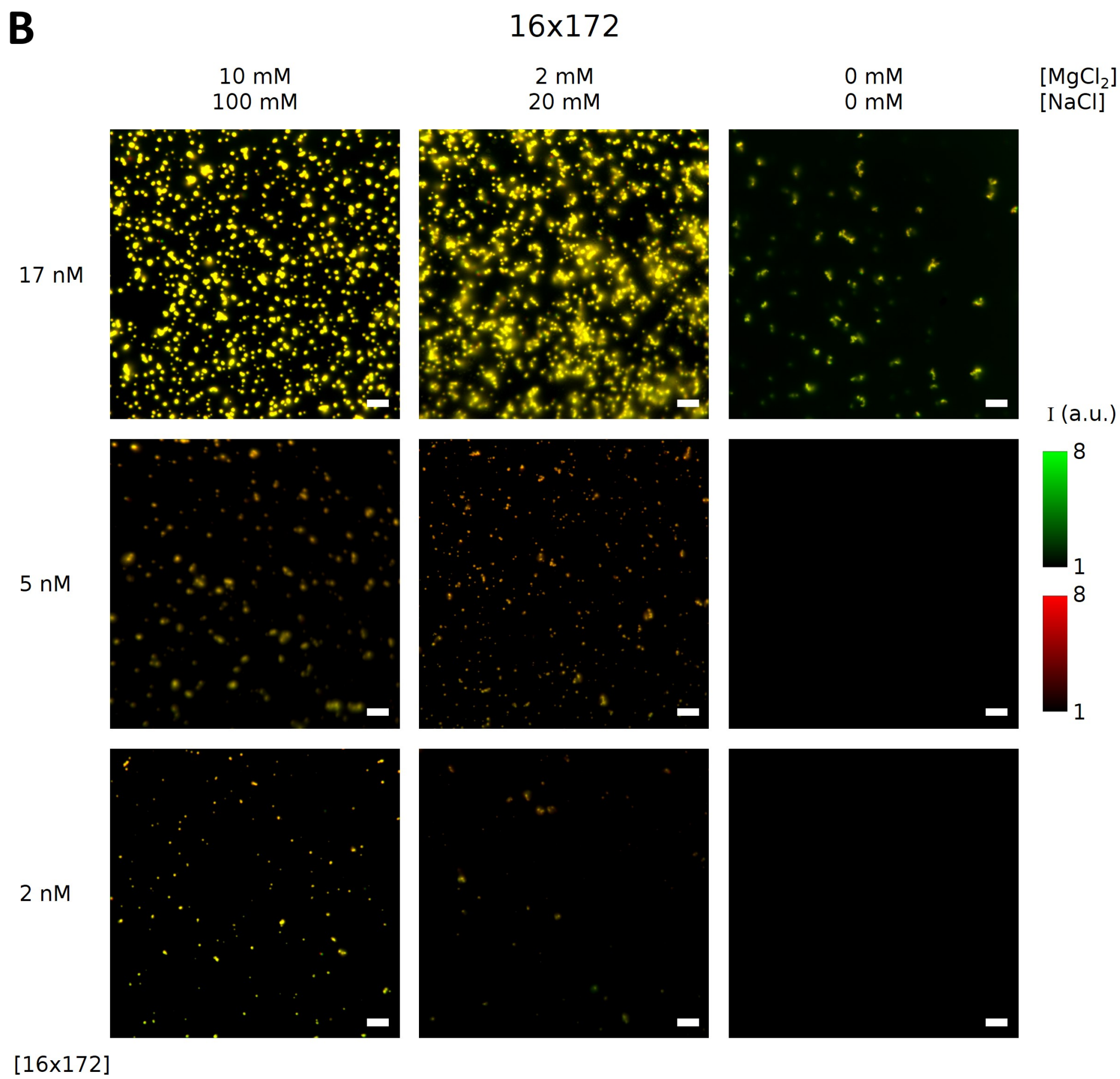
B
16x172
10 mM
100 mM
2 mM
20 mM
0 mM
0 mM
[MgCl2]
[NaCl]
17 nM
5 nM
2 nM
I (a.u.)
8
1
8
1
[16x172]

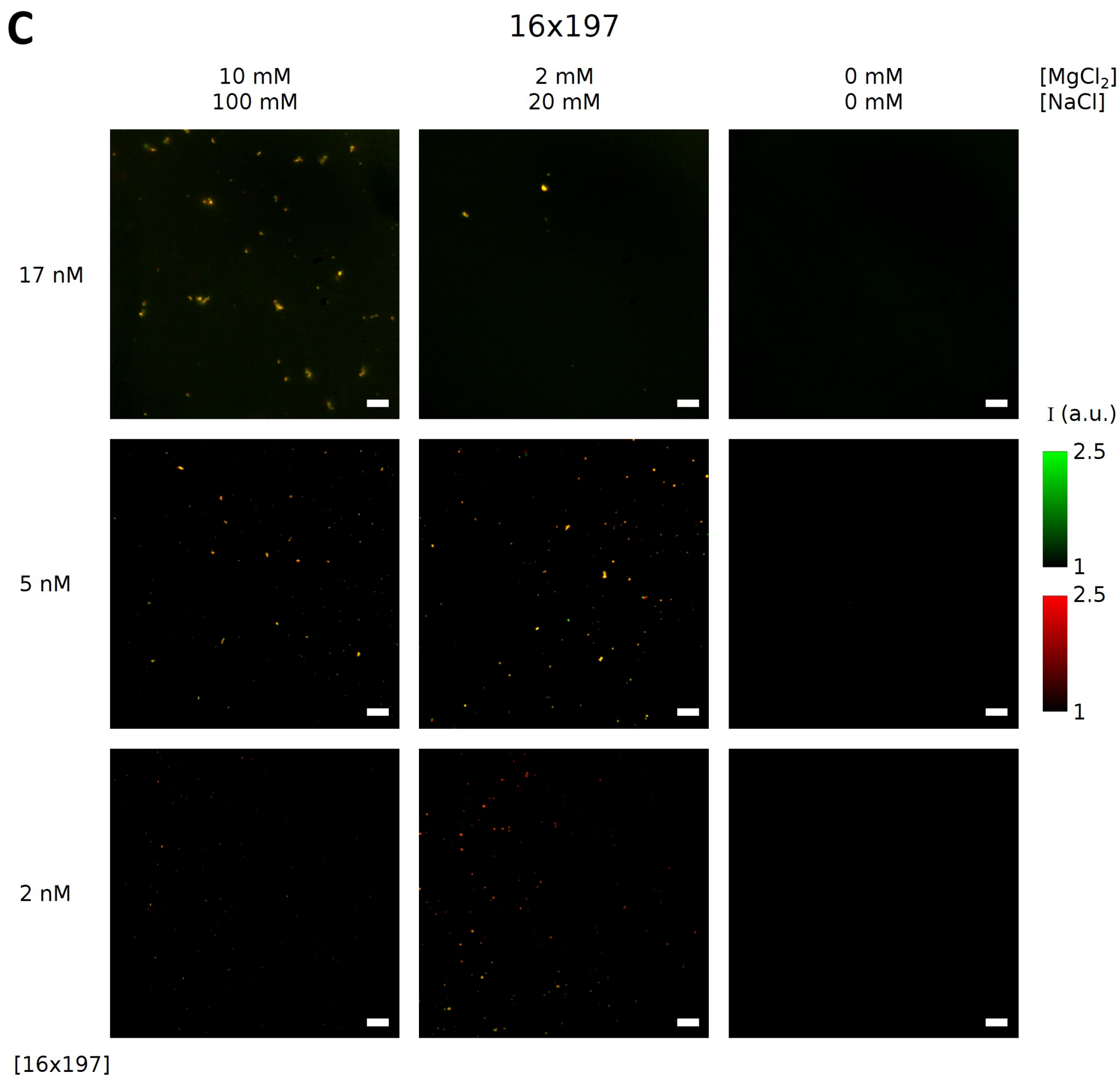

C
16x197
10 mM
100 mM
2 mM
20 mM
0 mM
0 mM
[MgCl2]
[NaCl]
17 nM
5 nM
2 nM
I (a.u.)
2.5
1
2.5
1
[16x197]

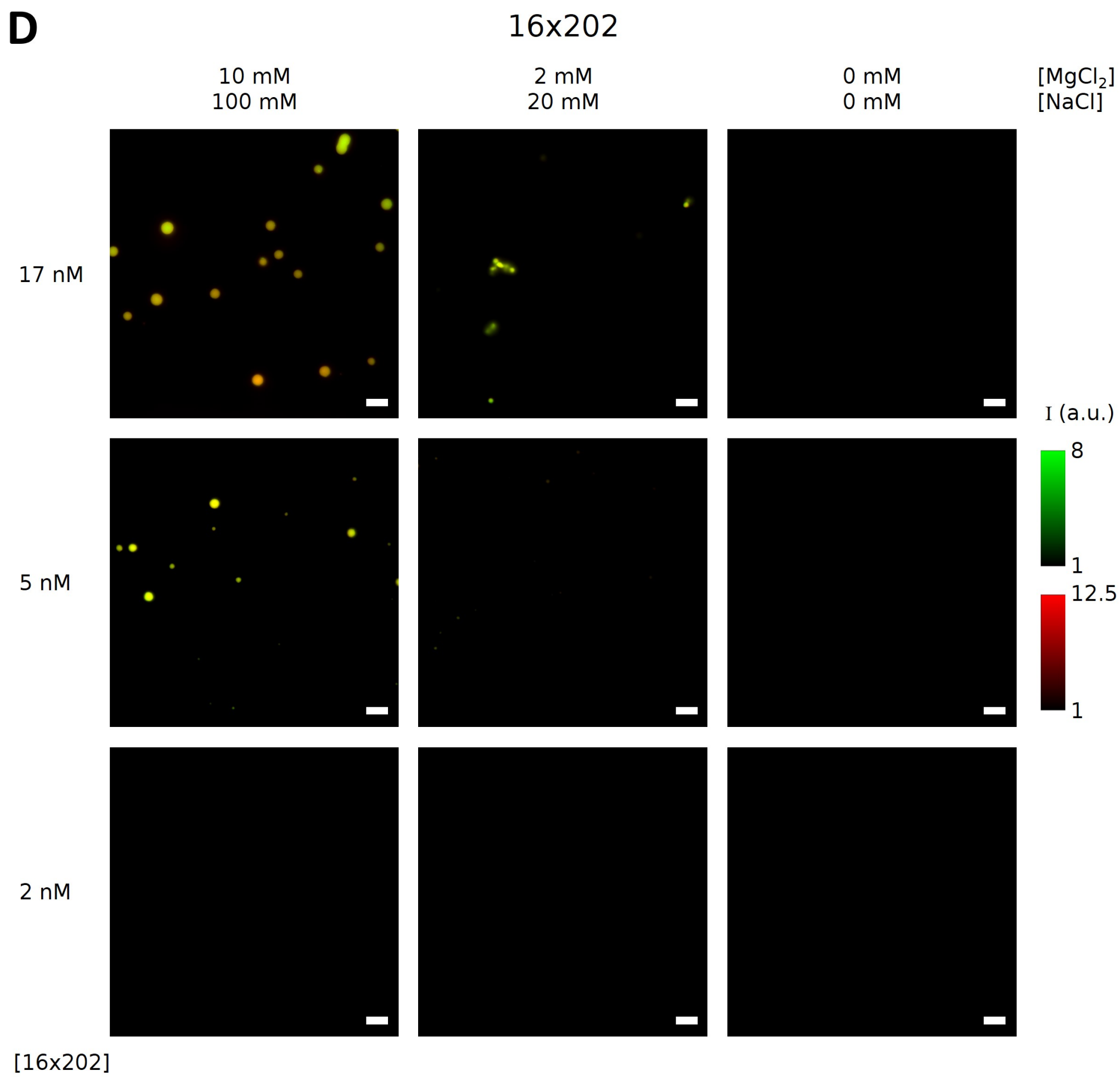

Figure S 4: **Phase diagrams of chromatin aggregation as a function of NRL. Chromatin fiber concentration and salt concentration are main driving factors in condensate formation. Condensate morphology is affected by NRL. A)** Condensates of 16 × 167 NRL fibers form amorphous aggregates under high NaCl, $MgCl_2$ and nucleosome concentration. **B)** Condensates of 16 × 172 NRL fibers form spherical condensates. **C)** Condensates of 16 × 197 NRL fibers form small (mostly < 1 µm) aggregates. **D)** Condensates of 16 × 202 NRL fibers form spherical condensates. Scale bar is 10 µm.

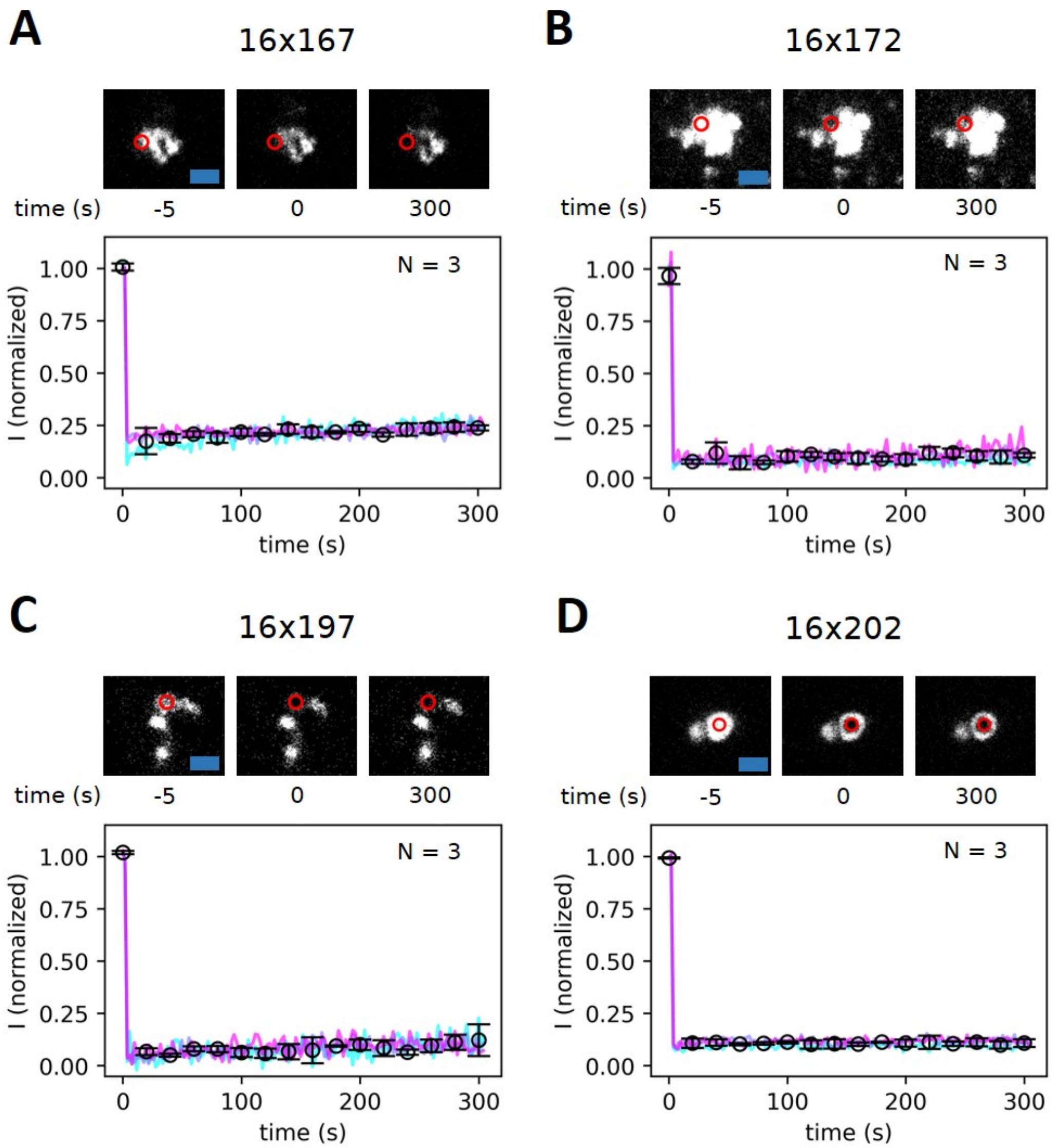

Figure S 5: **FRAP measurements on condensates 2 hours after inducing phase separation reveal no internal mobility, regardless of morphology.** Condensates formed with chromatin fibers of different linker lengths were partially bleached to investigate the mobility of chromatin fibers inside the condensate. Measurements were performed 2 hours after addition of phase separation buffer. A lack of signal recovery means limited or no mobility inside the condensate. **A)** FRAP measurement of condensates formed by 167 NRL fibers. Top - Representative condensates before (t = - 5 s), immediately after (t = 0 s) and 5 minutes after (t = 300 s) photobleaching. Red circle shows the photo-bleaching target area. Bottom - Mean normalized intensities of all traces, calculated every 20 s (black circles). N is the number of measured condensates. Errors are SD. Individual time traces are also shown (lines). No recovery is detected within the measurement time frame. **B)** Same as in A), but for 172 NRL. **C)** Same as in A), but for 197 NRL. **D)** Same as in A), but for 202 NRL. Scale bar is 2.5 µm.

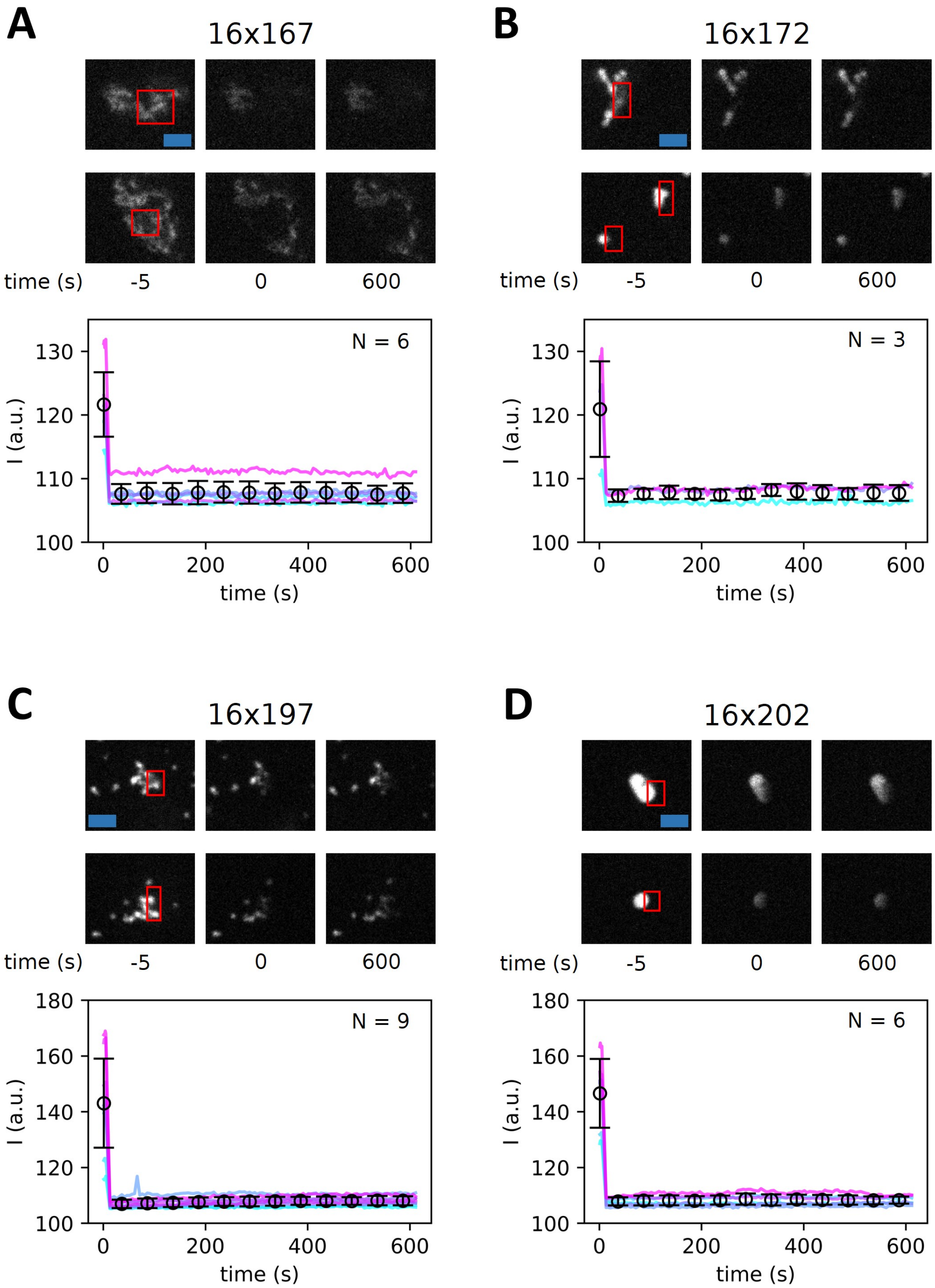

Figure S 6: **Internal mobility does not recover after inducing phase seperation.** FRAP measurements were performed 72 hours after the addition of the phase separation buffer. **A)** FRAP measurement of condensates formed by 167 NRL fibers. Top - Representative condensates before (t = - 5 s), immediately after (t = 0 s) and 10 minutes after (t = 600 s) photobleaching. Red square shows the photo-bleaching target area. Bottom - Mean intensities of all traces, calculated every 50 s (black circles). N is the number of measured condensates. Errors are SD. Individual time traces are also shown (lines). No recovery is detected within the measurement time frame. **B)** Same as in A), but for 172 NRL. **C)** Same as in A), but for 197 NRL. **D)** Same as in A), but for 202 NRL. Scale bar is 2.5 µm.

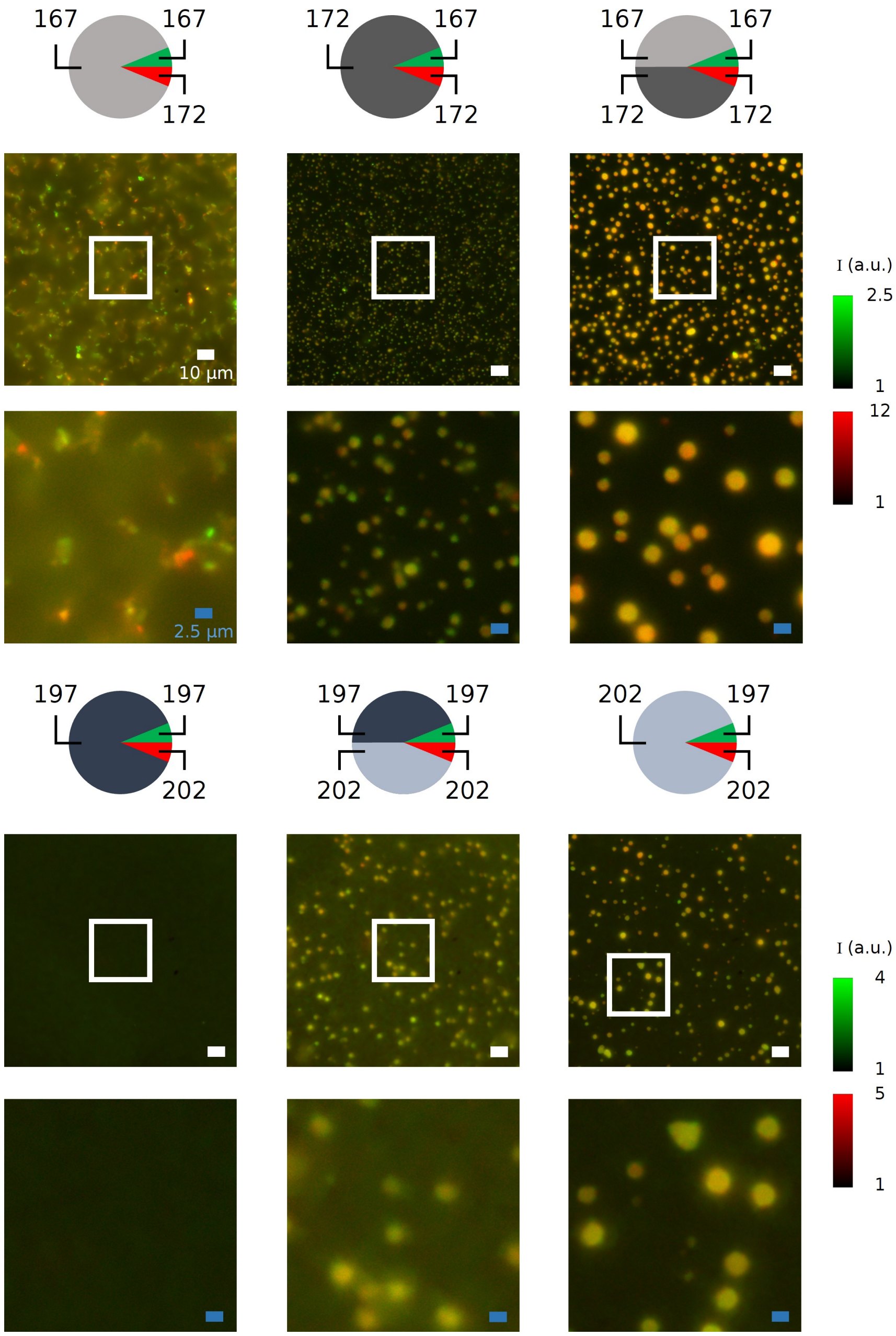

Figure S 7: **Full data used for Figure 3.** Top - schematic of chromatin linker lengths mixing ratios. Middle - Full FoV of measurement. Bottom - 4× magnified region of interest. The absence of condensates in the majority 197 NRL mixture (lower left) may reflect small differences in fiber and ionic concentrations which can further reduce the low concentration of condensates found in 197 NRL samples (Figure S4C). Scale bars are 10 µm (white) and 2.5 µm (blue).

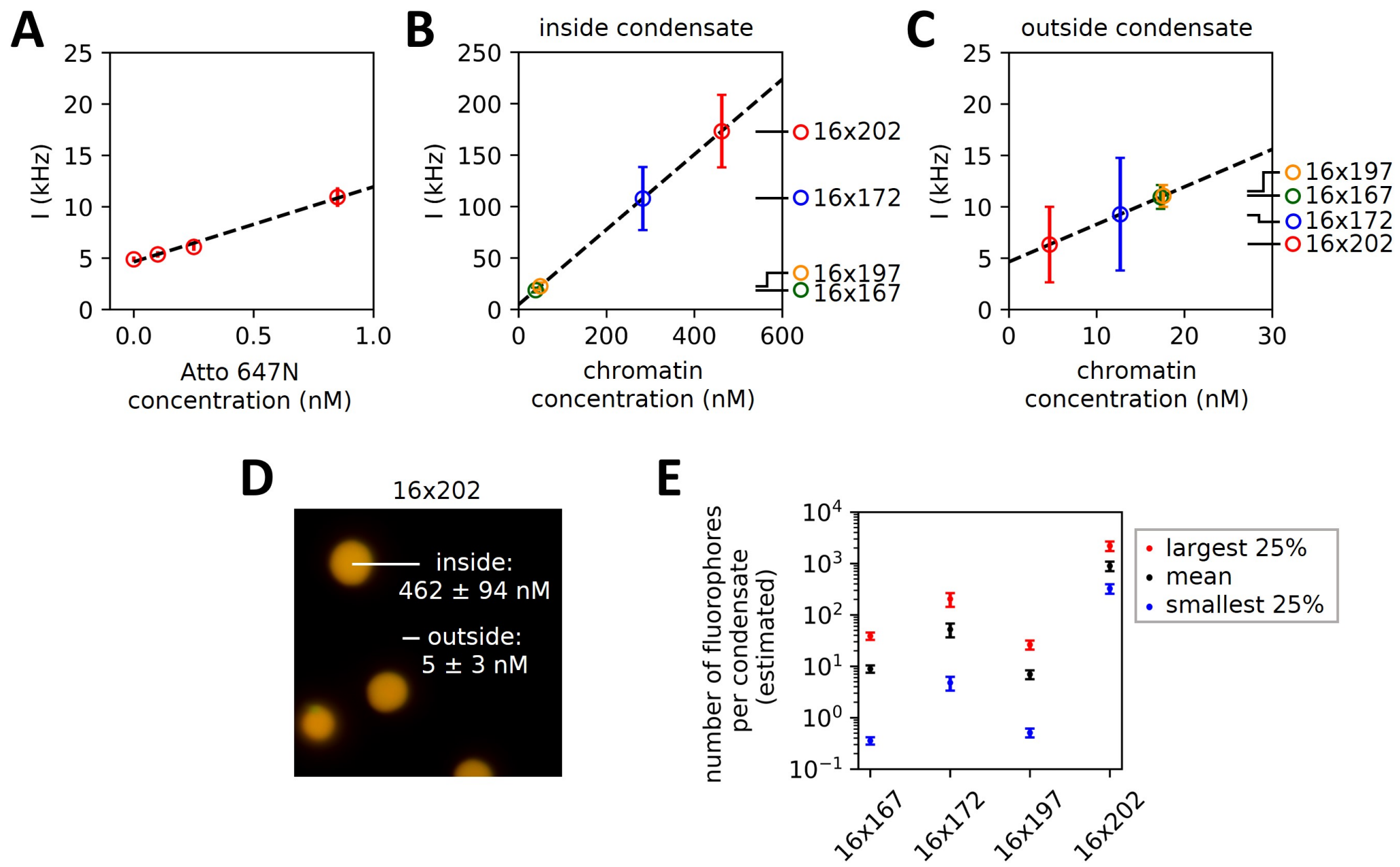

Figure S 8: **Quantification of chromatin concentration inside condensates and in solution. A)** Fluorescence signal intensity per pixel of samples with increasing Atto 647N concentration. Fitted curve is used to obtain the signal per Atto 647N molecule. **B)** Chromatin concentration inside 167 NRL (green), 172 NRL (blue), 197 NRL (orange) and 202 NRL (red) fiber condensates calculated from their mean fluorescence signal intensity per pixel (dotted line) and 1 in 20 labeling ratio. Slope of dotted line was extracted from A). Errors are SD between different condensates. **C)** as in B) but outside of the condensate. **D)** Visualization of the results for 202 NRL fiber condensates. **E)** Estimation of the number of fluorophores per condensate from previously calculated chromatin concentration and surface area. The volume of a condensate is assumed to be a sphere with cross-section area equal to that of the measured condensates. Errors are SD.

Plasmid insert oligonucleotide sequences

Inserts containing a BsaI site were created by annealing two complementary oligos. The overhangs are highlighted in **bold**.

Insert 1 oligo 1 (EcoRI + SapI site):

AATTCGTACAGCTATGAGACCAGCTGTTGCTCTTCC

Insert 1 oligo 2 (EcoRI + SapI site):

AGCGGAAGAGCAACAGCTGGTCTCATAGCTGTACG

Insert 2 oligo 1 (XbaI + NdeI site):

TATGCGGCAAGCTTTGCAGGTCGACTTGAAGGTCTCGTTACT

Insert 2 oligo 2 (XbaI + NdeI site):

CTAGAGTAACGAGACCTTCAAGTCGACCTGCAAAGCTTGCCGCA

Hairpin oligonucleotide sequences

The overhang is highlighted in **bold**. The hairpin is underlined. The position of the fluorophore label is shown in red (Atto 647N) or green (Cy3B)

Hairpin 1.1:

GCTAGACGACCATATGGTATGACCGTAATCGGTCATACCATATGGTCGTC

Hairpin 1.2 - labeled (Atto 647N, pos. 46):

GCTAGACGACCATATGGTATGACCGTAATCGGTCATACCATATGGTCGTC

Hairpin 2.1:

GTAAGACGACGCTGGGGTATGACCGTAATCGGTCATACCCCAGCGTCGTC

Hairpin 2.2 - labeled (Cy3B, pos. 36):

GTAAGACGACGCTGGGGTATGACCGTAATCGGTCATACCCCAGCGTCGTC

Quantification of DNA hairpin + T5 exonuclease reaction efficiency

fiber (16x)	167U	167G	167R	172U	172G	172R
start DNA (ug)	121	20	20	100	20	20
end DNA (ug)	84	11.1	9.5	69	10	11
yield (%)	69.4	55.5	47.5	69	50	55

fiber (16x)	197U	197G	197R	202U	202G	202R
start DNA (ug)	160	20	20	163	20	20
end DNA (ug)	110	12	12.35	122	13.6	12
yield (%)	68.8	60	61.8	74.8	68	60

Table S 1: Yields of hairpins + T5 exonuclease method. Samples are those shown in Figure S2 (U - unlabeled, G - labeled Cy3B, R - labeled Atto 647N)

Statistical significance of calculated parameters

fig.	name s1	parameter s1	name s2	parameter s2	p	p < 0.05
2	167U167G167R	area (um2)	172U172G172R	area (um2)	2.22E-01	
2	167U167G167R	area (um2)	197U197G197R	area (um2)	7.16E-02	
2	167U167G167R	area (um2)	202U202G202R	area (um2)	5.94E-05	TRUE
2	172U172G172R	area (um2)	197U197G197R	area (um2)	2.86E-01	
2	172U172G172R	area (um2)	202U202G202R	area (um2)	3.82E-05	TRUE
2	197U197G197R	area (um2)	202U202G202R	area (um2)	2.26E-05	TRUE
2	167U167G167R	circularity	172U172G172R	circularity	6.21E-81	TRUE
2	167U167G167R	circularity	197U197G197R	circularity	3.44E-05	TRUE
2	167U167G167R	circularity	202U202G202R	circularity	8.02E-47	TRUE
2	172U172G172R	circularity	197U197G197R	circularity	2.71E-08	TRUE
2	172U172G172R	circularity	202U202G202R	circularity	2.14E-06	TRUE
2	197U197G197R	circularity	202U202G202R	circularity	5.36E-11	TRUE
3	167U172R167G	S [all]	172U172R167G	S [all]	2.73E-17	TRUE
3	167U172R167G	S [all]	172U172R167U167G	S [all]	1.51E-26	TRUE
3	172U172R167G	S [all]	172U172R167U167G	S [all]	1.55E-16	TRUE
3	202U202R197G	S [all]	197U202R197G	S [all]	9.95E-01	
3	202U202R197G	S [all]	202U202R197U197G	S [all]	1.69E-24	TRUE
3	197U202R197G	S [all]	202U202R197U197G	S [all]	3.76E-01	
3	167U172R167G	S [largest 25%]	167U172R167G	S [smallest 25%]	8.59E-01	
3	172U172R167G	S [largest 25%]	172U172R167G	S [smallest 25%]	3.48E-02	TRUE
3	172U172R167U167G	S [largest 25%]	172U172R167U167G	S [smallest 25%]	2.22E-02	TRUE
3	202U202R197G	S [largest 25%]	202U202R197G	S [smallest 25%]	3.11E-01	
3	197U202R197G	S [largest 25%]	197U202R197G	S [smallest 25%]		
3	202U202R197U197G	S [largest 25%]	202U202R197U197G	S [smallest 25%]	1.59E-04	TRUE
4	167U167G_later172R	S [all]	172U172R_later167G	S [all]	8.33E-01	
4	167U167G_later172R	S [all]	167U167G_later172U172R	S [all]	2.08E-124	TRUE
4	172U172R_later167G	S [all]	167U167G_later172U172R	S [all]	9.44E-17	TRUE
4	167U167G_later172R	S [largest 25%]	167U167G_later172R	S [smallest 25%]	4.22E-01	
4	172U172R_later167G	S [largest 25%]	172U172R_later167G	S [smallest 25%]	1.80E-08	TRUE
4	167U167G_later172U172R	S [largest 25%]	167U167G_later172U172R	S [smallest 25%]	1.28E-02	TRUE
5	202U202R_later197G	S [all]	197U197G_later202R	S [all]	1.97E-01	
5	202U202R_later197G	S [all]	197U197G_later202U202R	S [all]	3.34E-01	
5	197U197G_later202R	S [all]	197U197G_later202U202R	S [all]	1.44E-01	
5	202U202R_later197G	S [largest 25%]	202U202R_later197G	S [smallest 25%]	6.08E-01	
5	197U197G_later202R	S [largest 25%]	197U197G_later202R	S [smallest 25%]		
5	197U197G_later202U202R	S [largest 25%]	197U197G_later202U202R	S [smallest 25%]		

Table S 2: p-values extracted from measurements shown in the results section, calculated using Welch's t-test. Sample names are each fiber NRL followed by its fluorescent label (U - unlabeled, G - labeled Cy3B, R - labeled Atto 647N). Where p-values are not given the mean ± SD of one of the parameters could not be calculated.
